# Tracing QTLs for Leaf Blast Resistance and Agronomic Performance of Finger Millet (*Eleusine coracana* (L.) Gaertn.) Genotypes through Association Mapping and *in silico* Comparative Genomics Analyses

**DOI:** 10.1371/journal.pone.0159264

**Published:** 2016-07-14

**Authors:** M. Ramakrishnan, S. Antony Ceasar, V. Duraipandiyan, K. K. Vinod, Krishnan Kalpana, N. A. Al-Dhabi, S. Ignacimuthu

**Affiliations:** 1 Division of Plant Biotechnology, Entomology Research Institute, Loyola College, Chennai, India; 2 Centre for Plant Sciences and School of Molecular and Cellular Biology, Faculty of Biological Sciences, University of Leeds, Leeds, United Kingdom; 3 Department of Botany and Microbiology, Addiriyah Chair for Environmental Studies, College of Science, King Saud University, Riyadh, Kingdom of Saudi Arabia; 4 Rice Breeding and Genetics Research Centre, Division of Genetics, ICAR-Indian Agricultural Research Institute, Aduthurai, India; 5 Regional Research Station, Tamil Nadu Agricultural University, Paiyur, India; 6 The International Scientific Partnership Program (ISPP) at King Saud University, Vice-Rectorate for Graduate studies and Research, Riyadh, Kingdom of Saudi Arabia; National Institute of Plant Genome Research (NIPGR), INDIA

## Abstract

Finger millet is one of the small millets with high nutritive value. This crop is vulnerable to blast disease caused by *Pyricularia grisea*, which occurs annually during rainy and winter seasons. Leaf blast occurs at early crop stage and is highly damaging. Mapping of resistance genes and other quantitative trait loci (QTLs) for agronomic performance can be of great use for improving finger millet genotypes. Evaluation of one hundred and twenty-eight finger millet genotypes in natural field conditions revealed that leaf blast caused severe setback on agronomic performance for susceptible genotypes, most significant traits being plant height and root length. Plant height was reduced under disease severity while root length was increased. Among the genotypes, IE4795 showed superior response in terms of both disease resistance and better agronomic performance. A total of seven unambiguous QTLs were found to be associated with various agronomic traits including leaf blast resistance by association mapping analysis. The markers, UGEP101 and UGEP95, were strongly associated with blast resistance. UGEP98 was associated with tiller number and UGEP9 was associated with root length and seed yield. Cross species validation of markers revealed that 12 candidate genes were associated with 8 QTLs in the genomes of grass species such as rice, foxtail millet, maize, *Brachypodium stacei*, *B*. *distachyon*, *Panicum hallii* and switchgrass. Several candidate genes were found proximal to orthologous sequences of the identified QTLs such as 1,4-β-glucanase for leaf blast resistance, cytokinin dehydrogenase (CKX) for tiller production, calmodulin (CaM) binding protein for seed yield and pectin methylesterase inhibitor (PMEI) for root growth and development. Most of these QTLs and their putatively associated candidate genes are reported for first time in finger millet. On validation, these novel QTLs may be utilized in future for marker assisted breeding for the development of fungal resistant and high yielding varieties of finger millet.

## Introduction

Finger millet (*Eleusine coracana* (L.) Gaertn.) is one of the small millets grown in different parts of the world including India. When compared to staple cereals such as wheat, rice and corn, finger millet has superior nutritional qualities [1]; it is one of the major constituents of cereal food supplements and health drinks. Known as *ragi* in India, this cereal occupies about 2.7 million hectares of cultivation worldwide especially in developing countries (Africa and Asia) with an annual production of 2.6 million tons with a contribution of about 10% of global millet production; 94% of the global finger millet production occurs in Africa and Asia [2]. This crop is highly vulnerable to leaf blast disease caused by *Pyricularia grisea* (teleomorph: *Magnaporthe grisea*) [3], and probably the only and most destructive disease occurring annually during rainy and winter seasons [2]. The disease occurs at all stages of plant growth more so as leaf, neck and finger blasts. Leaf blast is the initial one and most damaging. When the crop is severely affected by leaf blast, agronomic traits such as number of productive tillers, length of fingers, number of fingers and both yield and quality are severely affected [4]. It is estimated that average yield loss due to blast diseases is around 28–36% each year in Asia [5] and in certain areas yield losses can go as high as 80–100% [6].

Utilization of molecular marker based breeding approaches has been helpful for developing blast resistance and improving useful agronomic traits in rice and foxtail millet [7–9]. Many of these traits, including blast resistance, are under quantitative genetic control [10]. Genetic mapping of functional quantitative trait loci (QTLs) using molecular markers facilitates marker assisted breeding for crop improvement for the traits of interest [11–13]. Among different methods used in QTL mapping, association mapping (AM), also known as linkage disequilibrium (LD) mapping, offers more advantages for the dissection of complex genetic traits in plants [14, 15]. AM has much higher mapping resolution due to the use of natural, genetically diverse populations by targeting evolutionary recombinations that fragment chromosomes to very short but numerous haplotype blocks, over which marker-trait associations are identified [16, 17].

The AM approach has been applied widely as a tool for the identification of markers associated with useful agronomic traits in diverse plant species, including Arabidopsis [18, 19], maize [20], wheat [21], barley [22, 23], tomato [24], sorghum [25], rice [26, 27] and foxtail millet [8]. Except for few reports, such as identification of QTLs for agronomic traits including plant height, number of tillers, number of productive tillers, leaf length, seed yield [28, 29], protein and tryptophan contents in seeds [30] and leaf, neck and finger blast resistance [31], AM studies on finger millet have received less attention so far.

In view of the above, the present study was conducted using 128 finger millet genotypes sourced from various parts of the world to assess the phenotypic responses for leaf blast and its effect on useful agronomic traits. AM was performed to find useful QTLs, and the identified QTL information was used for *in silico* comparative genomics analysis with genomes of monocot model plants such as rice, foxtail millet, sorghum, maize, wheat, *Brachypodium* and switchgrass for the identification of candidate genes associated with QTLs. This study may lay the foundation for selection of genotypes and markers for finger millet breeding programmes to develop new varieties with improved agronomic traits in future.

## Materials and Methods

### Plant material and geographical region of the study

A test germplasm collection consisting of 128 finger millet genotypes originating from major diversity centers both in India and abroad, was assembled with the help of International Crops Research Institute for the Semi-Arid Tropics (ICRISAT), Patancheru, Hyderabad, India, University of Agricultural Sciences (UAS), Bangalore, India and Tamil Nadu Agricultural University (TNAU), Coimbatore, India. The details of the member genotypes and their origins were reported in our previous study [32]. These genotypes were assessed for their responses to leaf blast and other agronomic traits at Regional Research Station of TNAU located at Paiyur, Krishnagiri District, Tamil Nadu, which is one of the main areas of finger millet cultivation in India. Located at an elevation of 460 meters above sea level and at coordinates 12°25' N and 78°13' E, the chosen location has a long history of leaf blast prevalence during all seasons of finger millet cultivation and can be considered as a hotspot for blast disease.

### Experimental design

The experiment was laid out in fourteen blocks in an augmented design [33] having a block size of 9.45 m x 3 m during the month of March 2014. Three leaf blast susceptible varieties, RAU8, KM252 and HR374 and three leaf blast resistant varieties GPU28, CO14 and Paiyur2 were used as checks. In each block, nine test genotypes were grown along with all the checks. Each test accession was grown in three rows of 3 m length and each row consisted of 30 plants, while the checks were grown in two rows of 3 m length. The inter-row distance was 20 cm and plant to plant distance within row was 10 cm. The test genotypes were planted in alternate rows between check rows that were alternated with susceptible and resistant lines. In effect, there were 24 replications for each check. The design layout is provided in Figure A in S1 File. The plants were grown without blast disease management following the agronomic practices together with the nutrient supply of 60 kg/ha N, 30 kg/ha of P_2_O_5_ and 30 kg/ha of K_2_O recommended (http://agritech.tnau.ac.in/agriculture/millets_ragi.html) for finger millet cultivation.

### Evaluation for leaf blast resistance

Under field conditions, leaf blast disease was induced naturally and the symptoms developed on the leaves were monitored closely, measured and recorded after 45 days of sowing. A 0–5 score scale [34] was used for the assessment, for which ten randomly chosen plants per genotype were evaluated by scoring the affected leaves. The scores were, 0 –no symptoms on the leaves; 1 –small brown specks < 0.5mm diameter, no necrotic (collapsed cell) spots; 2 –slightly larger brown specks 2-3mm in diameter; 3 –round to elliptical lesions restricted up to 3mm in diameter with necrotic grey centre; 4 –typical elliptical blast lesion, restricted up to 6mm long with little coalescence of veins, yellow margin; and 5 –half or more of the leaf covered by coalescence of large lesions more than 6mm, yellowing, leaves may be killed by coalescence of large lesions. The score data were used for computing percent disease incidence (PDI) [35] with the formula:

$$\text{PDI}=\left(\frac{\text{Sum of numerical rating}}{\text{Total number of leaves observed x Maximum score}}\right)\;x\;100$$

The resistance and susceptibility of finger millet genotypes were assessed as per the method of Mackill and Bonnman [36], in which the plants were graded on a resistance scale of 0 to 5 as follows: highly resistant with 0% PDI (scale 0), resistant with 1% PDI (scale 1), moderately resistant with 1.1–5% PDI (scale 2), moderately susceptible with 5.1–25% PDI (scale 3), susceptible 25.1 to 50% PDI (scale 4) and highly susceptible with 50.1 to 100% PDI (scale 5). No occurrence of neck and finger blast was observed, and hence no observations were made on these symptoms.

### Evaluation of other agronomic traits

After 90 days of growth, the observations were made on eight important agronomic traits such as plant height, number of tillers, number of productive tillers, number of fingers, length of fingers, length of leaf and length of root. Seed yield was recorded at maturity after harvest. For each genotype, data were recorded from three random plants selected from each row and mean value was calculated. For the measurement of root length, the plants were extricated from soil after copious irrigation without damaging the root system and roots were washed with tap water and length was measured. Data were subjected to standard statistical analysis. To identify the direct relationship between leaf blast incidence and agronomic performance of the genotypes, the Pearson correlation was computed and a forward stepwise regression model was fitted to determine the most influential agronomic traits by leaf blast incidence, using PAST version 3.09 software [37].

### Cumulative ranking and grouping of genotypes based on phenotypic performance

For each of the agronomic trait, except for plant height, the genotype with highest phenotypic mean values was ranked first and all the remaining genotypes were ranked in descending order. For plant height, ideal plant height of 80 cm [38] was centred for ranking and deviants from the centred value were ranked both on ascending and descending order. For blast resistance, the genotype having the lowest PDI score was ranked first. The cumulative ranking (rank sum) of each of the genotype was computed by adding the ranks of that genotype for all eight agronomic traits. For instance, the genotype IE4795 had given rank 1 for blast resistance, rank 48.5 for plant height, rank 4.5 for tiller number, rank 8.5 for productive tiller number, rank 16 for finger number, rank 5 for finger length, rank 10 for leaf length, rank 71 for root length and rank 17 for seed yield. The rank sum was computed as 181.5. Similarly rank sums were computed for rest of the genotypes.

To have a better stratification of resistance and agronomic performance of the genotypes, an empirical classification of genotypes was done by grouping the resistance pattern as resistance (R) and susceptible (S), by taking 5% PDI as the cut-off point. Those genotypes with less than 5% PDI were classified as resistant and those exceeding 5% PDI were considered as susceptible. For the agronomic traits, the cumulative rank for agronomic traits alone was computed for each genotype ignoring the PDI rank in the computation. From this ranking top 10% of the total genotypes was selected as genotypes with good agronomic traits (GAT) and the rest were considered as genotypes with poor agronomic traits (PAT). By this way, four distinct groups of genotypes were obtained: R + GAT, R + PAT, S + GAT and S + PAT.

### Genotyping of finger millet genotypes

The genomic DNA was isolated from the test and check germplasms using a previously described protocol [39] with some modifications as reported earlier [40]. Genotyping was done using 87 genomic SSR markers with 60–70% GC content (Table A in S2 File) following the methods described previously [39] and 72 markers showed polymorphism. The population structure of the test and check germplasms were determined using 72 polymorphic markers as reported earlier [32].

### Population structure and kinship estimation

The population structure of the test and check germplasms was determined using the model-based Bayesian statistics implemented in the software STRUCTURE v.2.3.4 [41]. The number of subgroups (*k*) in the population was determined by running the programme by assuming *k* values ranging from 1 to 10, with five independent runs for each *k*. To fit the model, burn-ins were set to 100,000 for the Markov Chain Monte Carlo (MCMC) simulations [42] and data were collected over 500,000 MCMC replications in each run. The *k* value was detected by employing an *ad hoc* statistic Δ*K* based on the rate of change in the log probability of data between successive *k* values using Structure Harvester [43]. Inferred subpopulation coefficients (Q matrix) of individual genotype for the identified population structure were used as covariate in AM analyses. The genetic relatedness of the finger millets accessions was computed as kinship by weighing identical by state (IBS) of the common alleles among the genotypes [44] using the software TASSEL v5.2 [45]. The method scored the genotypes as 2, 1 or 0 equal to the count of one of the alleles at that locus. The missing genotypes were imputed by average genotype score. The relationship matrix was estimated from the score data. The method provided a better estimate of additive genetic variance.

### Association mapping

AM was performed using genotype data and mean phenotypic data including data on PDI and other 8 agronomic traits. The marker-trait association analysis was conducted using TASSEL v5.2 [45] employing both the general liner model (GLM) and mixed liner model (MLM) methods. The genotype data were formatted as numerical co-variates and used for analysis. In GLM method, the Q matrix comprising of inferred sub-population coefficients of the individuals was used as covariate in the model for suppressing the false discovery of marker trait association. Multiple testing corrections were implemented by adjusting marker probability values for multiple test runs, by running 1000 permutations. The significant association for a marker and trait was selected when the p value was below 0.005. To refine the results, in MLM method, along with the phenotypic data, kinship matrix (K matrix) was used as random effect in the model, in addition to the genotypic and Q matrix which were considered as fixed effects. The significant threshold for the association was set at *P* <0.01 for MLM method. Most common associations among the two approaches were taken as the valid marker-trait association.

### *In silico* comparative genomics

In the absence of detailed genome sequence information of finger millet, cross species validations of QTLs were attempted to identify any sequence similarity with validated markers, nucleotide using basic local alignment search tool (BLASTn). The original sequences of the random genomic libraries developed through *Hin*dIII, *Sal*I and *Pst*I digest and hybridization with probes from the finger millet accession PI 321125 used for designing the target markers (kindly provided by Dr Ketrien Devos, University of Georgia, College of Agricultural Science, USA) of identified QTLs associated with leaf blast resistance and agronomic traits [13] were used for *in silico* comparative genomics analysis. The sequences for BLASTn search were restricted to ten grass species available in Phytozome v11.0 (http://phytozome.jgi.doe.gov/pz/portal.html), an online web enabled tool. The positions of the sequence similarity found on the chromosome of the grass genomes were analyzed for the presence of any candidate gene near (upstream and downstream) the QTL sequence. The functions of any closely associated candidate gene were further analyzed for their relatedness to leaf blast resistance and other useful agronomic traits.

## Results

### Leaf blast assay

All finger millet genotypes germinated uniformly in the field within 4–5 days of sowing. The leaf blast incidence occurred in all the genotypes beginning from the seedling stage and became severe as the growth advanced. However, no significant incidence of neck and finger blast was observed in any of the genotype tested. Out of 128 genotypes tested, none of them was found to be highly resistant (scale 0) to leaf blast (Table B in S2 File); however, the leaf blast incidence varied significantly among the genotypes with PDI values ranging from 1.0% (IE4795) to 99.6% (HR374) with a mean of 33.0%. Besides IE4795, there were six genotypes such as CO14, GPU28, IE7079, IE4734, IE6165 and GPU67 with <5.0% PDI. Among these, one test genotype (IE4795) fell under resistant category (scale 1) and four genotypes, IE7079, IE4734, IE6165 and GPU67, were found to be moderately resistant (scale 2). The moderately resistant genotype IE6165 and IE7079 were sourced from Nepal and Kenya respectively. Rest of the genotypes was considered susceptible to leaf blast. Leaf blast resistant checks CO14 and GPU28 showed moderate resistance with PDI values of 3.2% and 4% respectively.

**Fig 1:**
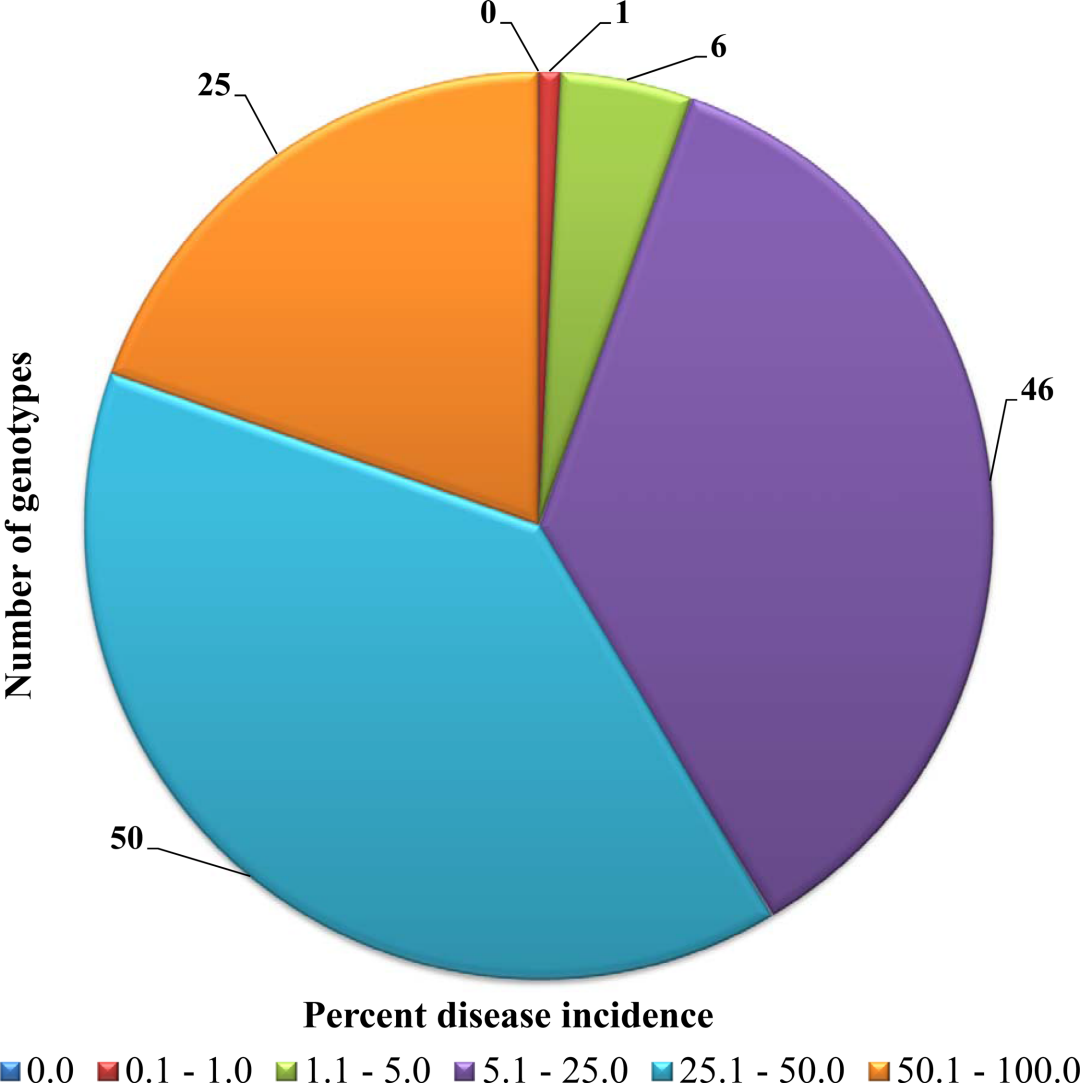
Leaf blast responses of 128 finger millet genotypes under field screening. No genotype was found highly resistant to leaf blast. Blast disease scores, 0: Highly resistant, 0.1–1.0: Resistant, 1.1–5.0: Moderately resistant, 5.1–25.0: Moderately susceptible, 25.1–50.0: Susceptible and 50.1–100: Highly susceptible.

Another leaf blast resistant check Paiyur2 showed moderate susceptibility with a PDI value of 11.2%. Of the rest, 46 genotypes were moderately susceptible (scale 3), 50 genotypes were susceptible (scale 4) and 25 genotypes were highly susceptible (scale 5) (Fig 1). The Nigerian genotype IE6537, Burundi genotype IE4709 and Senegal genotype IE5066 were found to be susceptible to leaf blast. The leaf blast susceptible check, HR374 (PDI 99.6%) was the most susceptible genotype in the experiment, followed by check genotypes RAU8 (PDI 81.6%) and KM252 (PDI 70.4%). Among the test genotypes, INDOF5 (PDI 76.8%), KM301 (PDI 73.6%), Vijayawada (PDI 71.2%) and INDOF8 (PDI 69.6%) were highly susceptible.

### Agronomic performance of genotypes

Despite severe leaf blast all the genotypes survived to maturity, and significant level of phenotypic variations was observed for all agronomic traits (Table 1). The plant height ranged from 34.3–110.3 cm with an average of 75.3 cm. The genotypes IE4646, CO14, MR6, IE2589 and HR374 had the ideal plant height of 80.0, 80.3, 80.5, 80.5 and 80.6 cm respectively, while GPU67 (34.3 cm) and HR911 (40.0 cm) were the shortest genotypes. There were 76 genotypes that produced mean plant height of less than 80 cm. The number of tillers varied between 1 and 5 per plant with a mean of 2.3 tillers. The genotype IE3470 produced average of maximum 5 tillers per plant, whereas INDOF5, APSSK1, IE5870, L5 and THRVP produced one tiller per plant. There were 1–4 productive tillers per plant among the 128 genotypes tested, of which the genotypes IE3470 and IE2911 produced average of 4 productive tillers per plant. The genotypes INDOF7, IE5091, TCHIN1 and IE5817 showed poor tillering response and produced unproductive tillers bringing down the average below 1 (Table B in S2 File).

**Table 1:** Spectrum of phenotype variation and leaf blast response among 128 genotypes of finger millet.

Traits	Mean	Range	CV %	Standard Error
Plant height (cm)	75.32	34.3–110.3	22.90	1.52
Number of tillers / plant	2.25	1.0–5.0	31.90	0.06
Number of productive tillers / plant	1.73	0.3–4.0	42.27	0.06
Number of fingers / head	5.11	1.0–9.3	33.98	0.15
Length of fingers (cm)	5.54	1.7–10.1	32.66	0.16
Length of leaf (cm)	30.04	16.3–46.3	20.83	0.55
Length of root (cm)	17.12	11.0–25.3	15.18	0.23
Total seed yield (gm)	2.62	0.9–8.4	49.35	0.11
PDI (%)	33.01	1.0–99.6	60.10	1.75

PDI, Percent disease incidence; CV, coefficient of variation

The number of fingers per head varied from 1–9.3, averaging 5.1 fingers. The genotype IE3104 produced superior response for number of fingers with an average of 9.3, whereas IE5817 and IE2457 produced only one finger per head. Average finger length observed in the population was 5.5 cm, which varied between 1.7 (IE5817) to 10.1 cm (HR374). Among the test genotypes, leaf length varied between 16.3 and 46.3 cm, with an average leaf length of 30.0 cm. The genotype IE2957 produced the longest leaf (46.3 cm), while HR911 had the shortest leaf.

The average root length of the genotypes was 17.1 cm that ranged from 11.0 cm to 25.3 cm. The genotype SVK1 produced roots of 25.3 cm length followed by IE2572, IE2619 and IE2042 with roots measuring 24.4, 23.3 and 23.2 cm respectively. The genotypes GPU67 (11.0 cm), IE2710 (11.7 cm) and IE6082 (11.8 cm) showed inferior response for root length. There was significant variation in seed yield among the genotypes ranging between 0.9 gm to 8.4 gm per plant. The average seed yield among the population was 2.6 gm. The genotype RAU8 produced superior response with 8.4 gm of mean seed yield per plant. This was followed by GPU67 which produced 8.0 gm of mean seed yield per plant. The genotype IE2619 produced 0.9 gm of mean seed per plant (Table 1).

### Interrelations of agronomic traits and leaf blast incidence

Phenotypic relations between agronomic traits and leaf blast incidence (PDI) indicated that number of tillers had a negative relation with PDI, while root length had a positive relation. Plant height exhibited positive association with number of tillers, length of fingers and root length. Similarly, number of tillers was correlated with number of productive tillers and leaf length. Number of productive tillers indicated positive association with number of fingers and finger length. Number of fingers had a positive correlation with finger length while it was negatively correlated with leaf length. Length of fingers was the only trait that showed a significant association with seed yield. Since there are many auto-correlated traits in the phenotype data, to establish the significant factors that can be associated to disease incidence, a forward stepwise multiple regression analysis was performed (Table 2). The analysis revealed that only two traits such as plant height and root length were significantly associated with leaf blast incidence, wherein plant height had a negative influence on PDI (-0.25) while the length of root had a significant positive influence on PDI (1.78).

**Table 2:** Forward stepwise multiple regression analysis between leaf blast incidence and agronomic traits.

Parameter	Estimate	Standard Error	T- Statistic	P-Value	Adj. R^2^ (%)
CONSTANT	21.67	12.51	1.73	0.09	6.54
Plant height (cm)	-0.25	0.10	-2.49	0.01	
Length of root (cm)	1.78	0.67	2.64	0.01	

Durbin-Watson statistic = 1.204 (P = 0.00)

### Cumulative ranking and classification

**Fig 2:**
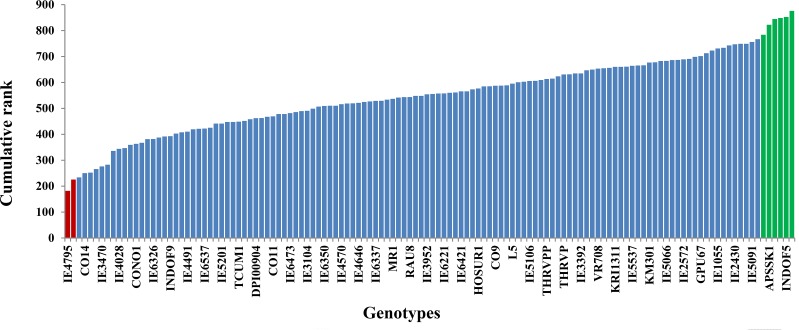
Cumulative ranks for all the traits for 128 genotypes of finger millet. The data were collected after 45 and 90 days of sowing the seeds for leaf blast and agronomic traits respectively. Superior and inferior genotypes are represented by green bars and red bars respectively.

Based on the total rank for all traits, the genotype IE4795 was found to be a superior genotype (Fig 2), combining best agronomic performance with leaf blast resistance. This was followed by CO14 and GPU28 which were ranked second and third for overall performance. Both of these genotypes were used as the blast resistant checks in this trial.

**Fig 3:**
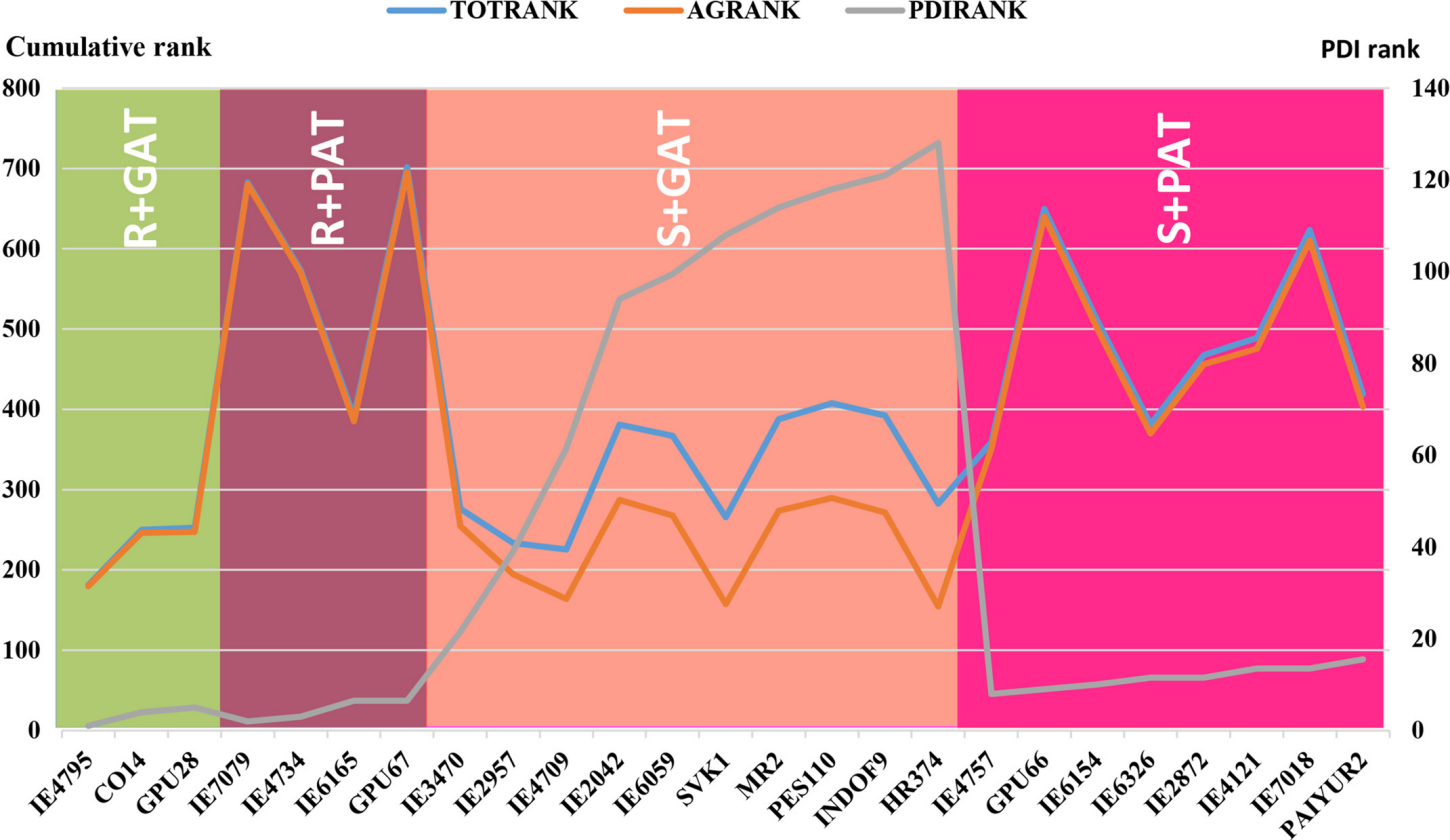
Empirical classification of top 25 ranking finger millet genotypes based on cumulative rank sum of agronomic traits and disease incidence percent; R+GAT, R+PAT, S+GAT and S+PAT. The remaining 103 genotypes belonged to the last category S+PAT.

The empirical classification brought out the spectrum of genotypes based on their overall performance by giving equal emphasis to disease reaction and agronomic performance. Out of 128 genotypes, 7 genotypes were found to be better genotypes for leaf blast resistance, while 13 were found with good agronomic traits at 10% selection. There were four groups; the resistance coupled with good agronomic traits (R+GAT) group had three genotypes (IE4795, CO14 and GPU28); the resistance with poor agronomic traits (R+PAT) group had four genotypes (IE7079, IE4734, IE6165 and GPU67); the susceptible with good agronomic traits (S+GAT) group had ten genotypes (IE3470, IE2957, IE4709, IE2042, IE6059, SVK1, MR2, PES110, INDOF9 and HR374) and the susceptible with poor agronomic traits (S+PAT) group had 111 genotypes (Fig 3).

### Association mapping

AM revealed that 15 QTLs (markers) were associated with seven traits by GLM method and seven QTLs were associated with five traits by MLM method. All the seven QTLs identified in MLM were concordant to GLM method. By GLM method, three QTLs each were identified for number of productive tillers and seed yield and two QTLs each for leaf blast resistance, number of tillers, number of fingers and length of root, while one QTL was identified for plant height. There were two QTLs identified for leaf blast resistance, associated with the markers UGEP101 and UGEP95 reporting 21.05 and 8.95% of phenotypic variation for PDI respectively. Both these QTLs were discovered by MLM method too, with corresponding R^2^ values of 6.07 and 5.79% respectively.

By GLM method, number of tillers and number of productive tillers were associated with two common markers UGEP98 and UGEP65, of which UGEP98 was also identified in MLM method which explained 9.97% of phenotypic variation for number of tillers. As that of leaf blast resistance, two QTLs (UGEP9 and UGEP57) identified for root length by GLM method were also confirmed using MLM method, explaining a phenotypic variation of 8.12 and 6.28% respectively. For seed yield, by MLM method only one QTL, UGEP9 (R^2^ = 7.57%) was found to be associated among the three identified by GLM method. UGEP9 was also found to be associated with root length. Only one marker UGEP50 was associated with plant height by GLM method, which was not identified by MLM method (Table 3).

**Table 3:** The details of genomic SSR markers associated with leaf blast resistance and agronomic traits using GLM and MLM methods in 128 genotypes of finger millet.

Trait	Locus	GLM (p<0.005)		MLM (p<0.01)	
		p- value	R^2^ (%)	p- value	R^2^ (%)
Leaf blast resistance	UGEP101	0.000	21.05	0.006	6.07
	UGEP95	0.001	8.95	0.008	5.79
Plant height	UGEP50	0.003	6.69	-	
Number of tillers	UGEP98	0.000	9.53	0.000	9.97
	UGEP65	0.001	11.72	-	
Number of productive tillers	UGEP98	0.002	7.00	0.006	6.55
	UGEP65	0.003	7.00	-	
	SSR01	0.004	6.51	-	
Number of fingers	UGEP104	0.003	6.85	-	
	UGEP75	0.004	6.31	-	
Length of root	UGEP9	0.001	8.12	0.002	8.12
	UGEP57	0.005	6.28	0.006	6.28
Seed yield	UGEP9	0.000	10.71	0.008	7.57
	UGEP19	0.001	7.63	-	
	UGEP80	0.003	6.78	-	

### *In silico* comparative genomics

The flanking sequences of microsatellite regions associated with fourteen out of the fifteen identified QTLs were observed to be orthologous to the genomic regions of the grass species such as rice (*Oryza sativa*), foxtail millet (*Setaria italica*), maize (*Zea mays*), *Brachypodium distachyon*, *B*. *stacei*, *Panicum hallii* and switchgrass (*Panicum virgatum*). The algorithm parameters such as score value, identity and E-value ranged from 39.2 to 461, 64.4 to 100% and 0.1 to 8.00E-128 respectively (S1 Table). The hits ranged between 1 and 427 in a species per QTL. The markers UGEP101, UGEP50, UGEP65, UGEP98, UGEP104, UGEP9, UGEP57 and UGEP19 were found proximally associated with 12 candidate genes reported in seven grass species (Table 4). However, there were no direct hits on the candidate gene sequences *per se*. The marker, UGEP57 obtained maximum number of hits of 895, followed by UGEP50, UGEP75, UGEP95, UGEP65 and UGEP104 with total hits of 682, 387, 369, 356, and 347 respectively. Multiple hits on the same chromosome were also observed for the marker UGEP50 that displayed maximum of 56 hits on chromosome 1 in maize. Significant hits were not found for the markers, UGEP101, UGEP50, UGEP98 and UGEP65 in *B*. *distachyon*, UGEP101 and UGEP98 in *B*. *stacei*, UGEP101 in *P*. *hallii*, UGEP95 in rice and UGEP65 in maize.

**Table 4:** The details of the putative candidate genes identified on other grass species genomes, proximal to the orthologous regions for the finger millet QTLs by *in silico* comparative genomics analysis. The function of each gene is also indicated.

Marker	Species	Linkage group	Number of hits	E-Value range	Locus name	Distance from the marker (kb)	Candidate Gene	Known functions with references
UGEP101	*Zea mays*	Chr 3	10	1.8E-5	GRMZM2G130864_T01	21.82 (DS)	1,4-β-Glucanase	Fungal resistance [46]
UGEP50	*Setaria italica*	Scaffold _3	9	1.9E-3	Seita.3G298400.1	74.11 (DS)	Cytochrome P450 CYP2	Plant growth [47]
UGEP65	*Oryza sativa*	Chr 1	2	4.5E-3	LOC_Os01g10110.1	29.41 (US)	CKX	Tiller growth and yield [48]
UGEP98	*Panicum hallii*	Chr 6	8	7.0E-2	Pahal.F00786.1	3.49 (DS)	ARF	Tiller growth and development [49]
UGEP104	*Brachypodium distachyon*	Chr 1	5	1.0E-47	Bradi1g38238.1	58.45 (US)	ERF	Flower development [50]
		Chr5	5	2.8E-4	Bradi5g11270.1	1.26 (US)	MADS TF	Meristem determinacy and development [51]
	*Panicum virgatum*	Chr 3a	44	2.2E-46	Pavir.Ca00579.1	82.64 (US)	MADS box protein	Inflorescence development [52]
	*Setaria italica*	Scaffold_7	8	6.1E-2	Seita.7G132800.1	26.66 (US)	ZF-C2H2_6	Trichome development on the inflorescence [53]
UGEP9	*Setaria italica*	Scaffold_4	8	6.5E-2	Seita.4G278800.1	6.29 (DS)	Ser/Thr Protein kinase	Enhances early root growth and development [54]
	*B*. *stacei*	Chr 5	2	3.7E-2	Brast05G258700.1	51.92 (DS)	Cytochrome P450 CYP2	Primary root growth and development [55]
UGEP57	*Oryza sativa*	Chr 4	12	7.2E-41	LOC_Os04g49730.1	13.20 (DS)	PMEI	Primary root growth and development [56]
UGEP19	*Oryza sativa*	Chr10	8	7.4E-7	LOC_Os10g28420.1	62.00 (DS)	CaM -binding protein	Regulating Ca^2+^ signaling in plants [57]

US, upstream; DS, downstream; CKX, Cytokinin dehydrogenase; TF, transcription factor; ARF, Auxin response factor; ERF, Ethylene-responsive transcription factor; PMEI, Pectin methylesterase inhibitor; ZF, Zinc finger; CaM, Calmodulin.

**Fig 4:**
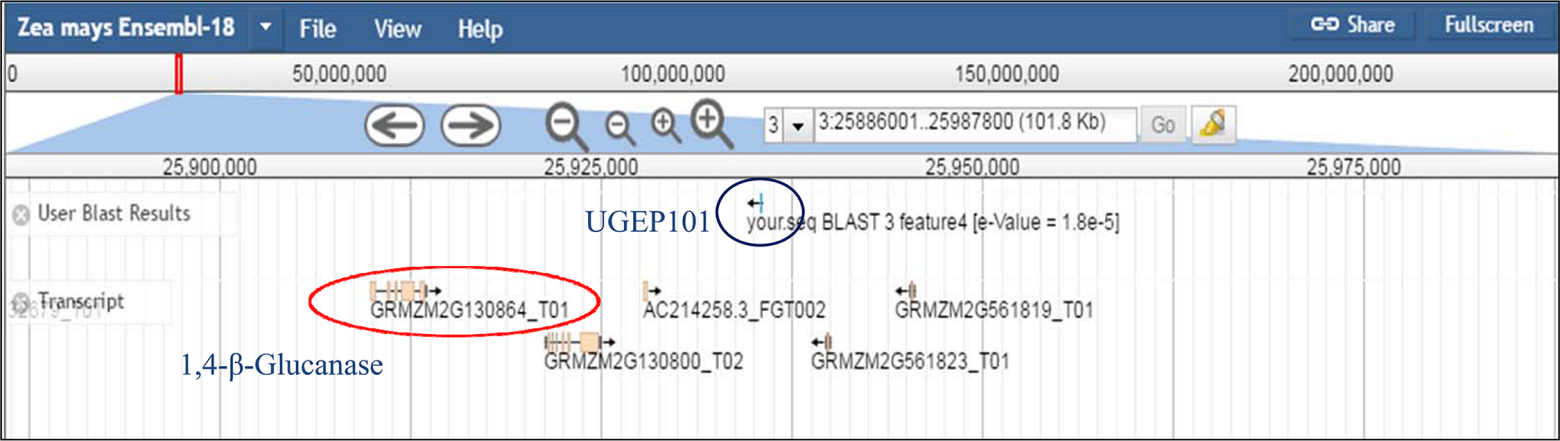
Screen shot image of comparative genomics analysis with QTL UGEP101 in genome of maize. UGEP101 is associated with candidate gene 1, 4-Beta-Glucanase at 21.822 kb distances in maize chromosome 3; this gene is responsible for fungal resistance trait.

**Fig 5:**
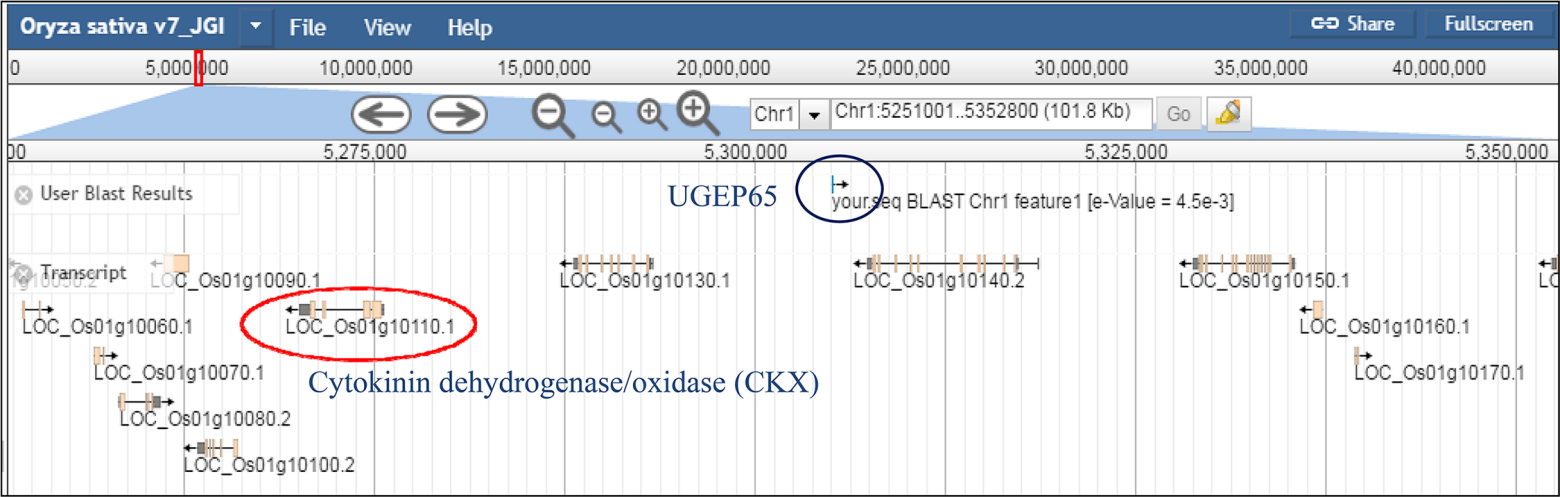
Screen shot image of comparative genomics analysis with QTL UGEP65 in genome of rice. UGEP65 is associated with candidate gene CKX at 29.412 kb distance in rice chromosome 1; this gene is responsible for tiller growth and seed yield trait.

The putative candidate genes identified included a 1,4-β-glucanase gene at 21.8 kb downstream proximity to the UGEP101 orthologue on the chromosome 3 in maize with an E-value of 1.8E-5 (Fig 4). A cytochrome P450 CYP2 gene was located 74.1 kb downstream from the UGEP50 orthologue at scaffold-3 in foxtail millet, with an E value of 1.9E-3 (Figure B in S1 File). Orthologous sequences for the marker UGEP65, were found proximal to a cytokinin dehydrogenase (CKX) gene located 29.4 kb upstream (US) on the rice chromosome 1 (Fig 5). Further, a pectin methylesterase inhibitor (PMEI) gene and calmodulin (CaM)-binding protein gene were located 13.2 kb downstream from the UGEP57 orthologue on rice chromosome 4 (Figure C in S1 File) and 62.0 kb downstream from UGEP19 orthologue on rice chromosome 10 (Figure D in S1 File) respectively. The 3.5 kb downstream sequences from the orthologous region for the marker UGEP98 on *Panicum hallii* genome contained an auxin response factor (ARF) gene localised on the chromosome 6 (Figure E in S1 File). Orthologous sequences to the marker UGEP104 identified on *Brachypodium distachyon* genome, was closer to an ethylene-responsive transcription factor (ERF) located 58.5 kb away upstream on chromosome 1 (Figure F in S1 File) and a MADS transcription factor at 1.26 kb upstream on chromosome 5 (Figure G in S1 File) respectively. Sequences similar to the marker UGEP104, was identified on the switchgrass (*Panicum virgatum*) genome 82.64 kb upstream of a MADS box protein on chromosome 3a (Figure H in S1 File) and 26.66 kb upstream of a zinc finger (ZF)-C2H2_6 on the foxtail millet genome scaffold_7 (Figure I in S1 File). Orthologues for UGEP9, were located near a Ser/Thr protein kinase gene on the foxtail millet Scaffold-4 6.29 kb downstream (Figure J in S1 File) and 51.92 kb downstream of a cytochrome P450 CYP2 gene on the chromosome 5 of the *Brachypodium stacei* genome (Figure K in S1 File).

## Discussion

### Blast occurrence and genetic resistance

The natural field used in the present study was known to be a hotspot for leaf blast disease in finger millet [58]. None of the genotypes was found to be highly resistant, in contrast to an earlier report [59] of natural field screening of 190 genotypes where some genotypes were found to be highly resistant. Artificial leaf blast screening in the greenhouse identifies significantly higher number of highly resistant genotypes [31]. Screening under natural field condition could be a better option than greenhouse screening, because genotypes with enduring resistance may not be identified under artificial conditions. Among the resistant checks used in the present study, CO14 and GPU28 showed moderate resistance to leaf blast, reinforcing the fact that field level evaluation is a better scale of comparison for enduring blast resistance. Further, this was supported by high susceptibility shown by all susceptible checks included. This has confirmed that natural field used in this study was optimal for leaf blast screening. The experimental design that alternated resistant and susceptible checks on either side of every test genotype provided uniform disease load throughout the crop duration. Comparative screening has helped to identify one genotype, IE4795, which showed resistance to leaf blast coupled with best agronomic performance. Further, screening under natural field conditions was very helpful to find the level of resistance exhibited by different genotypes and to classify them into different categories based on resistance.

Pathotypes of *Magnaporthe oryzae* are known to show distinct host adaptation, genetic diversity and sexuality [60] and hence it is likely that they may show distinct adaptive variation in North and South Indian conditions that are agro-climatically distinct. Earlier, Nagaraja et al. [61] assessed field incidence of blast disease under different agro climatic conditions at Bangalore, Vizianagaram and Ranichauri and reported that low temperature, high relative humidity (>80%) and high rainfall were conducive for blast development especially the neck and finger blasts. Under the arid weather conditions with moderate high temperature at the present study location, leaf blast incidence was found to supersede neck and finger blasts. Further, young plants were more susceptible to leaf blast than mature plants, indicating the plasticity of the blast pathotypes to adapt to changing environments and crop phenology. To assess the blast reaction under South Indian conditions, 82 genotypes from ICRISAT collection were included in our study, that were also tested earlier at Almora, Uttarakhand, North India, a known blast hotspot for both finger millet and rice [31]. Besides, an additional 46 genotypes were also included in this study that were unexplored with respect to their genetic diversity and leaf blast resistance.

Spectrum of leaf blast reaction among the 128 genotypes revealed very low proportion of resistance in the population (<1.0%), which was lower than that reported from an earlier study [31], in which 12 were resistant genotypes among 190 genotypes. This led us to hypothesise the possibility that blast pathogen at the hotspot location of Paiyur could be genetically adapted to the region and distinct from the pathotype found in North Indian conditions. Reinforcing this theory, we found that leaf blast resistance spectrum of common genotypes shared between both the studies showed largley contrasting reactions. Eleven resistant genotypes reported earlier [31] were found susceptible or moderately susceptible/resistant under this study, while the resistant genotype IE4795 identifiied in this study was reported moderately resistant at North Indian conditions. A parellel situation was also encountered in the case of moderate resistance, wherein the proportion of genotypes was significanly low in the studied population. None of the four moderately resistant genotypes identified in this study was reported to show better resistance under North Indian conditions. Significant variation in blast reactions among common genotypes tested under different agro-geographical regions were reported earlier in finger millet [58] and rice [62].

Blast disease in finger millet is manifested as leaf, neck and finger blasts, of which interrelationships among the different manifestations are not well established. Although leaf blast is reported to affect seedlings, and less severe compared to other forms, no clear relation between the leaf and head infections is reported [63]. However, when neck and finger blasts are established, they could significantly affect agronomic traits [31, 59] than the leaf blast. We have not found any incidence of neck and finger blasts in this study. Nevertheless, we have observed significant adverse association of leaf blast with agronomic traits. Although finger and neck blasts can create more adverse influence on agronomic traits over leaf blast, under favourable conditions, leaf blast can also incite equally damaging influence on number of productive tillers, number and length of fingers and grain yield [5, 61]. Hence when established alone, leaf blast can become effectively destructive and cause reduction of plant height and root length in finger millet genotypes. There are no previous reports available on the leaf blast screening in different finger millet genotypes at Paiyur in South India. This study clearly indicates that agro-climatic conditions play a major role in leaf blast incidence in finger millet and the spectrum of genotypes showing varying levels of resistance to leaf blast can be utilized in identifying genetic regions that govern resistance and utilize the resistance sources for marker assisted breeding programmes in future.

### Agronomic performance under leaf blast load

The agronomic performance of the genotypes was relatively poor under the present study wherein the genotypes were widely infected with leaf blast and left unmanaged. As done in the case of blast incidence assessment, the performance of genotypes that were common to previous studies provided us an opportunity to compare the agronomic performance of such genotypes. In the present study, the genotype IE4646 produced optimum mean plant height of 80.0 cm [38] followed by the USA genotype IE2589 (80.5 cm). Both these genotypes were reported to produce maximum plant height of 93.0 cm and 137.0 cm respectively across diverse agro-ecological situations in India [31]. This could be attributed to their high susceptibility to leaf blast in the present study, the probable reason for their poor performance. Corroborating this theory, the resistant genotype IE4795 was found to produce plant height of 106 cm, similar to the height of 105.2 cm reported earlier [31]. Similar observations were also recorded for other agronomic traits, such as number of productive tillers that ranged between 0.3–4.0 in the current study as against 1–18 under well managed conditions [29]. Reports on direct influence of leaf blast susceptibility on agronomic performance of finger millet genotypes are scanty in literature. Cross species reports on the influence of blast disease on agronomic performance are available especially in rice, wherein significant influence of blast in reducing the plant height and number of productive tillers has been described [64]. McRae [65] who reported finger millet blast disease for the first time in India, estimated losses to a tune of 50%; however, there are later reports of loss up to 90% under severe infections [66].

The multiple regression analysis of agronomic traits on leaf blast incidence revealed significant relation of plant height and root length to PDI. While plant height reduced with severity of infection, root length increased. Leaf blast occurs in susceptible plants from early stages severely crippling the growth due to adverse influence on physiological health of the plants including poor photosynthetic activity of the affected leaves resulting in reduced plant height. Under leaf blast infection, significant reduction of net photosynthetic rate of the affected leaves is reported in rice [66]. Further, the infection itself puts the plants into severe stress and nutrient depletion. The increased elongation of the roots therefore can be attributed to the adaptive mechanism of the plant to forage for more food and nutrients when aerial part undergoes severe stress due to the disease. No previous reports are available for correlating leaf blast with root length in finger millet. The genotype IE4795 stood first in consolidated rank and produced superior response for disease resistance and other agronomic traits. Assessment of interrelations of agronomic traits in the present study revealed that positive correlation was observed for plant height, number of tillers, length of fingers and length of root. Upadhyaya et al. [67] reported a positive correlation for plant height and length of fingers in 622 genotypes of finger millet.

Based on empirical classification, only three genotypes (IE4795, CO14 and GPU28) can be recommended for cultivation under leaf blast endemic areas of South India where agroclimate similar to Paiyur exist. These genotypes were grouped under R + GAT category that combined resistance with good agronomic traits. IE4795 is a finger millet accession from Zimbabwe belonging to ICRISAT minicore collection [67]. This genotype was reported to be resistant to neck and finger blast at Almora in North India [68]. IE4795 was reported to be admixture genotype of Indian and African populations for finger millet having more than 50% of alleles from Indian population [28]. CO14 is a high yielding finger millet variety of South India, developed from the pedigree Malawi 1305/ CO 13 with an average yield of 2774 kg/ha. These genotypes were reported to be moderately resistant to neck and finger blast [69]. GPU28 is a highly blast resistant variety developed from the pedigree Indaf 5/ IE 1012 with an average yield of 3500–4000 kg/ha from Karnataka, India (http://www.nuscommunity.org/resources/our-publications/publication/recommended-package-of-agro-production-technology-for-finger-millet/).

Ten genotypes were identified under S+ GAT category having good agronomic traits despite of being susceptible to the disease. These genotypes need to be improved for leaf blast resistance. They can be used as the recurrent parents in a marker assisted finger millet improvement program. Also they can be used as donor lines for best agronomic trait. However, there were four genotypes under R + PAT category that combined resistance but with poor agronomic traits; these genotypes could be used as donor lines for leaf blast resistance. One hundred and eleven genotypes were grouped into S+ PAT category, which neither had resistance nor agronomic superiority; these genotypes require improvement in disease resistance, which may improve their agronomic performance. Further, under leaf blast endemic situations such varieties may require intensive disease management to realise better yield. Some of these genotypes can also be used as susceptible checks in blast resistance breeding in finger millet.

### Detection of QTLs

The marker-trait association with blast resistance and other agronomic traits clearly demonstrated that use of SSR markers was successful in deciphering QTLs for these traits in the present study. SSR markers are the next best alternative for mapping especially in self-pollinated crops like finger millet wherein evolutionary haplotype blocks may be larger when compared to cross-pollinated species [70, 71]. Further, SSRs are multi-allelic and therefore may offer better testing opportunity for the association between complex phenotypic traits and candidate locus, because single-loci SNP analyses may present a loss of information due to the bi-allelic nature [72]. Seven out of fifteen QTLs concordant in both GLM and MLM methods of association mapping are considered robust and unambiguous in the present study. MLM method includes an additional co-variate of kinship so that false discovery of association is greatly regulated than in GLM method, because there is an effective control for population structure and relatedness within genome-wide association studies [15].

Successful use of SSR markers in association analyses for agronomic traits and disease resistance have been demonstrated earlier in finger millet [28, 31, 68] and in rice [73]. We have identified two novel QTLs linked to markers UGEP101, and UGEP95 associated for leaf blast incidence among the genotypes that can be related directly to resistance. Both these markers have so far not been assigned to any linkage group [74]. Earlier studies have [31] identified three QTLs associated with markers such as FMBLEST35, FMBLEST15 and RM23842 for leaf blast resistance using genic SSR markers. However, relationship if any, among these markers, could not be ascertained in the absence of a linkage map involving these markers.

Three agronomic traits, tiller number (both total and productive), root length and seed yield were found associated with markers UGEP98, UGEP9 and UGEP57. None of these markers had been assigned to finger millet linkage groups so far [13, 74]. UGEP98 was not so far reported to be associated with tiller number in finger millet. The earlier reported marker UGEP81 associated with basal tiller number [28] was not found to be associated with tiller number in the present investigation. The marker-trait associations identified in this study have not been reported earlier and are considered novel as far as the current knowledge goes. Most of these markers were synthesized from random genomic libraries of the finger millet accession PI 321125 [13]. In the absence of linkage information, we are unable to determine the proximity of the identified markers to previously reported markers.

### Identification of putative candidate genes linked to QTLs

The evolutionary cascade of grass genomes showcases several conserved genetic regions, genome wide spread over 10000 grass species [75]. Taking advantage of this, we have followed QTLs to candidate gene tracking using comparative genomics approach, in which identified QTLs were associated with important candidate genes, using marker sequences. This method was used as an alternate attempt in the absence of genome information for finger millet. The basis of the search was to find any cross genome syntenous regions targeting specific traits that may also contain similar SSR sequences proximal to the candidate genes. The SSR sequences from the amplified fragments were not used because the sequences themselves may not be appropriate for realising significant hits because of the ubiquitous nature of tandem repeats as well as due to the fragment length variation within and between genomes; hence we have used the original flanking sequences of the microsatellite regions associated with QTLs for BLASTn search. Although this has helped to identify some candidate genes associated with these markers, we resort not to claim their authenticity pending validation. This approach differed from previously reported approach [30, 31, 76], in which markers were designed from candidate gene sequences from related genera such as rice and associated with the trait of interest. Recently, foxtail millet genome has been sequenced among the small millets [77, 78, 79], which may help in identifying trait related candidate genes for finger millet in the future.

Out of the twelve putative candidate genes identified from grass genomes proximal to the orthologous regions to the eight finger millet QTL linked markers, the gene 1,4-β-glucanase was significant as it was found adjoining to the leaf blast resistance orthologue in maize genome. 1, 4-β-glucanase gene has been identified to play a major role in fungal resistance in major cereal species including rice [46] and wheat [80]. QTL linked marker for plant height UGEP50 was orthologous to foxtail millet sequences found adjacent to a cytochrome P450 CYP2 gene. Cytochrome P450 CYP2 superfamily is known to be essential for internode elongation, plant growth [47, 81] and primary root growth [55]. Similarly, UGEP65, the marker linked to the number of tillers was found to be putatively associated to the cytokinin dehydrogenase/oxidase (CKX) gene in rice, the down regulation of which has been identified as important for tiller number and grain yield [48]. Positional cloning of the QTL *Gn1a* that is associated to the spikelet number in rice was identified to encode *OsCKX2*, which controls the rice grain yield by regulating cytokinin accumulation through reduced expression of *Gn1a* in the inflorescence meristems resulting in increased spikelet number [82]. The auxin response factor (ARF) gene essential for tiller growth and development identified in the *Panicum hallii* genome [49] was aanother UGEP65 associated putative candidate gene in the present study.

The finger development in finger millet is a flowering process that is under the influence of several flowering related genes that are found to be associated to the orthologous sequences of the finger development QTL linked marker UGEP104 across genomes of *Brachypodium distachyon*, switchgrass and foxtail millet. The genes such as ethylene response factor (ERF), minichromosome maintenance protein 1 (MCM1)-Agamous-Deficiens-serum response factor (SRF) transcription factor (MADS-TF), MADS box protein, zinc finger—Cys2His2 (ZF-C2H2) are regulatory genes essential for flower development [50], meristem determinacy [51], inflorescence development [52] and trichome development on the inflorescence [53] respectively. Therefore, putative association of more than one candidate genes to a locus makes it difficult to recommend a specific functionality to such locus. Further, the primary root growth and development were found suggestively linked to candidate genes Ser/Thr protein kinase [54], cytochrome P450 CYP2 [55] and pectin methylesterase inhibitor (PMEI) [56] by their association to the orthologous sequences to the markers UGEP9 and UGEP57 linked to root length in finger millet.

The seed yield related marker UGEP9 in finger millet was found proximal to a CaM binding protein gene on the rice orthologue on chromosome 10. CaM binding proteins are reported to be important for regulating Ca^2+^ signaling in plants [57]. A Ca^2+^ regulating protein, a seed dominant CaM was reported to be essential for calcium accumulation in finger millet grains [83]. Finger millet is the richest source of grain calcium among the millets [84], and the role of calcium in determining seed yield [85] is very crucial.

We have identified several cross genome orthologues to the identified markers linked to various traits in finger millet, that were proximal to several candidate genes that are known to regulate the respective traits in other grass species. However, there are no previous reports on the role of these candidate genes in finger millet. Therefore, these genes require independent validation for their role in finger millet genome.

## Conclusion

Finger millet accessions in this study showed significant variation in resistance to leaf blast disease that had greatly altered their agronomic performance. However, we could identify few potential cultivars that combine resistance and agronomic performance which can be used directly for cultivation as well as donors for useful genes in finger millet improvement. Although the lack of whole genome information remains as a major impediment in designing molecular marker based crop breeding in finger millet today, microsatellite marker based genome scans as performed in this study can provide great leads in this direction. Recent advances in whole genome information in foxtail millet spearheaded by the next generation sequencing (NGS) technology [79] also can accelerate identification of candidate genes in finger millet. In view of this, the sequencing of finger millet genome/transcriptome can result in development of high-throughput markers which would advance finger millet genomics in the same way as it happened to foxtail millet. Further investigations on the identified varieties can help in locating novel genomic regions associated with agronomic performance and disease resistance; also the use of identified QTLs can lead us to develop new efficient cultivars for the future.

## Supporting Information

S1 File **Figures A-K, (A) The design layout of the experiment.** The test genotypes were grown alternated with resistant and susceptible checks to provide adequate disease load throughout cropping season; (**B) Screen shot image of comparative genomics analysis with QTL UGEP50 in genome of foxtail millet**. QTL UGEP50 was associated with candidate gene Cytochrome P450 CYP2 at 74.112 kb distances in foxtail millet Scaffold-3 for internode length and plant growth; **(C) Screen shot image of comparative genomics analysis with QTL UGEP57 in genome of rice**. QTL UGEP57 was associated with candidate gene PMEI at 13.201 kb distance in rice chromosome 4 for primary root growth; **(D) Screen shot image of comparative genomics analysis with QTL UGEP19 in genome of rice**. QTL UGEP19 was associated with candidate gene CaM-binding protein at 61.899 kb distance in rice chromosome 10 for calcium accumulation in finger millet grains; **(E) Screen shot image of comparative genomics analysis with QTL UGEP98 in genome of hall’s panicgrass.** QTL UGEP98 was associated with candidate gene ARF at 3.491 kb distances in hall’s panicgrass chromosome 6 for tiller growth and development; **(F) Screen shot image of comparative genomics analysis with QTL UGEP104 in genome of *Brachypodium distachyon*.** QTL UGEP104 was associated with candidate gene ERF at 58.488 kb distances in *Brachypodium distachyon* chromosome 1 for Flower development; (**G) Screen shot image of comparative genomics analysis with QTL UGEP104 in genome of *Brachypodium distachyon*.** QTL UGEP104 was associated with candidate gene MADS transcription factor at 1.256 kb distances in *Brachypodium distachyon* chromosome 5 for Meristem determinacy and development; **(H) Screen shot image of comparative genomics analysis with QTL UGEP104 in genome of switchgrass.** QTL UGEP104 was associated with candidate gene MADS box protein at 82.637 kb distances in switchgrass chromosome 3a for Inflorescence development; **(I) Screen shot image of comparative genomics analysis with QTL UGEP104 in genome of foxtail millet.** QTL UGEP104 was associated with candidate gene ZF-C2H2_6 at 26.657 kb distances in foxtail millet Scaffold_7 for Trichome development on the inflorescence. **(J) Screen shot image of comparative genomics analysis with QTL UGEP9 in genome of foxtail millet.** QTL UGEP9 was associated with candidate gene Ser/Thr protein kinase at 6.292 kb distances in foxtail millet Scaffold_4 for early root growth and development; **(K) Screen shot image of comparative genomics analysis with QTL UGEP9 in genome of *Brachypodium stacei*.** QTL UGEP9 was associated with candidate gene Cytochrome P450 CYP2 at 51.917 kb distances in *Brachypodium stacei* chromosome 5 for Primary root growth and development.
(PDF)

S2 File **Tables A-B, (A) Agronomic performance and percentage of disease incidences (PDI) of the 128 finger millet genotypes;** the data were collected after 45 and 90 days of sowing the seeds for PDI and agronomic traits respectively; **(B) List of 87 genomic SSR primers with primer sequence (5’ to 3’)**, SSR motif and size of markers (bp) used for the analysis of population structure of 128 finger millet genotypes.
(PDF)

S1 Table The details of the algorithm parameters such as score value, identity and E-value for each QTL as analyzed in 10 model crops by *in silico* comparative genomics. File contains separate sheet for each QTL.
(XLSX)

## References

[1] Mirza N, Sharma N, Srivastava S, Kumar A. Variation in Popping Quality Related to Physical, Biochemical and Nutritional Properties of Finger Millet Genotypes. Proc Natl Acad Sci, India, Sect B Biol Sci. 2014:1–9.

[2] Nagaraja A, Kumar J, Jain AK, Narasimhudu Y, Raghuchander T, Kumar Bijendar, et al Compendium of small millets diseases AICSMIP, UAS, GKVK, Bangalore 2007:1–80.

[3] Ignacimuthu S, Ceasar S. Development of transgenic finger millet (*Eleusine coracana* (L.) Gaertn.) resistant to leaf blast disease. J Biosci. 2012;37:135–47. 22357211 10.1007/s12038-011-9178-y

[4] Pall BS. Assessment of loses due to neck blast of ragi (*Eleusine coracana* (L) Gaertn) Food farming Agric. 1977;9:55.

[5] Nagaraja A, Jagadish PS, Ashok EG, Gowda KTK. Avoidance of finger millet blast by ideal sowing time and assessment of varietal performance under rainfed production situations in Karnataka. J Mycopath Res 2007;45:237–40.

[6] Rao ANS. Estimates of losses in finger millet (*Eleusine coracana*) due to blast disease (*Pyricularia grisea*) J Agri Sci. 1990;24:57–60.

[7] Tabien R, Li Z, Paterson A, Marchetti M, Stansel J, Pinson S. Mapping QTLs for field resistance to the rice blast pathogen and evaluating their individual and combined utility in improved varieties. Theor Appl Genet. 2002;105:313–24. 12582534 10.1007/s00122-002-0940-2

[8] Gupta S, Kumari K, Muthamilarasan M, Parida S, Prasad M. Population structure and association mapping of yield contributing agronomic traits in foxtail millet. Plant Cell Rep. 2014;33:881–93. 10.1007/s00299-014-1564-0 24413764

[9] Vanniarajan C, Vinod KK, Pereira A. Molecular evaluation of genetic diversity and association studies in rice (*Oryza sativa* L.). Journal of Genetics. 2012;91:9–19. 22546822 10.1007/s12041-012-0146-6

[10] Fu YB. Understanding crop genetic diversity under modern plant breeding. Theor Appl Genet. 2015;128:2131–42. 10.1007/s00122-015-2585-y 26246331 PMC4624815

[11] Cadic E, Coque M, Vear F, Grezes-Besset B, Pauquet J, Piquemal J, et al Combined linkage and association mapping of flowering time in Sunflower (*Helianthus annuus* L.). Theor Appl Genet. 2013;126:1337–56. 10.1007/s00122-013-2056-2 23435733

[12] Neumann K, Kobiljski B, Denčić S, Varshney RK, Börner A. Genome-wide association mapping: a case study in bread wheat (*Triticum aestivum* L.). Mol Breeding. 2011;27:37–58.

[13] Dida M, Srinivasachary, Ramakrishnan S, Bennetzen J, Gale M, Devos K. The genetic map of finger millet, *Eleusine coracana*. Theor Appl Genet. 2007;114:321–32. 17103137 10.1007/s00122-006-0435-7

[14] Mackay I, Powell W. Methods for linkage disequilibrium mapping in crops. Trends in Plant Science. 2007;12:57–63. 17224302 10.1016/j.tplants.2006.12.001

[15] Zhang Z, Ersoz E, Lai C-Q, Todhunter RJ, Tiwari HK, Gore MA, et al Mixed linear model approach adapted for genome-wide association studies. Nat. Genet. 2010;42:355–60. 10.1038/ng.546 20208535 PMC2931336

[16] Gupta P, Rustgi S, Kulwal P. Linkage disequilibrium and association studies in higher plants: Present status and future prospects. Plant Mol Biol. 2005;57:461–85. 15821975 10.1007/s11103-005-0257-z

[17] Upadhyaya H, Wang Y-H, Gowda CLL, Sharma S. Association mapping of maturity and plant height using SNP markers with the sorghum mini core collection. Theor Appl Genet. 2013;126:2003–15. 10.1007/s00122-013-2113-x 23649651

[18] Atwell S, Huang YS, Vilhjálmsson BJ, Willems G, Horton M, Li Y, et al Genome-wide association study of 107 phenotypes in a common set of *Arabidopsis thaliana* inbred lines. Nature. 2010;465:627–31. 10.1038/nature08800 20336072 PMC3023908

[19] Brachi B, Faure N, Horton M, Flahauw E, Vazquez A, Nordborg M, et al Linkage and Association Mapping of Arabidopsis thaliana Flowering Time in Nature. PLoS Genetics. 2010;6:e1000940 10.1371/journal.pgen.1000940 20463887 PMC2865524

[20] Guo B, Beavis W. In silico genotyping of the maize nested association mapping population. Mol Breeding. 2011;27:107–13. 10.1007/s11032-010-9503-4 PMC3015163 21289856

[21] Joukhadar R, El-Bouhssini M, Jighly A, Ogbonnaya FC. Genome-wide association mapping for five major pest resistances in wheat. Mol Breeding. 2013;32:943–60.

[22] Wang M, Jiang N, Jia T, Leach L, Cockram J, Waugh R, et al Genome-wide association mapping of agronomic and morphologic traits in highly structured populations of barley cultivars. Theor Appl Genet. 2012;124:233–46. 10.1007/s00122-011-1697-2 21915710

[23] Cockram J, White J, Zuluaga DL, Smith D, Comadran J, Macaulay M, et al Genome-wide association mapping to candidate polymorphism resolution in the unsequenced barley genome. Proc Natl Acad Sci U S A. 2010;107:21611–6. 10.1073/pnas.1010179107 21115826 PMC3003063

[24] Mazzucato A, Papa R, Bitocchi E, Mosconi P, Nanni L, Negri V, et al Genetic diversity, structure and marker-trait associations in a collection of Italian tomato (*Solanum lycopersicum* L.) landraces. Theor Appl Genet. 2008;116:657–69. 10.1007/s00122-007-0699-6 18193185

[25] Wang M, Zhu C, Barkley N, Chen Z, Erpelding J, Murray S, et al Genetic diversity and population structure analysis of accessions in the US historic sweet sorghum collection. Theor Appl Genet. 2009;120:13–23. 10.1007/s00122-009-1155-6 19760215

[26] Jin L, Lu Y, Xiao P, Sun M, Corke H, Bao J. Genetic diversity and population structure of a diverse set of rice germplasm for association mapping. Theor Appl Genet. 2010;121:475–87. 10.1007/s00122-010-1324-7 20364375

[27] Agrama HA, Eizenga GC, Yan W. Association mapping of yield and its components in rice cultivars. Mol Breeding. 2007;19:341–56.

[28] Babu BK, Agrawal PK, Pandey D, Jaiswal JP, Kumar A. Association mapping of agro-morphological characters among the global collection of finger millet genotypes using genomic SSR markers. Mol Biol Rep. 2014;41:5287–97. 10.1007/s11033-014-3400-6 24861452

[29] Bharathi A. Phenotypic and genotypic diversity of global finger millet (*Eleusine coracana* (L.) Gaertn.) composite collection. PhD thesis-TNAU-Coimbatore. 2011.

[30] Babu BK, Agrawal PK, Pandey D, Kumar A. Comparative genomics and association mapping approaches for opaque2 modifier genes in finger millet accessions using genic, genomic and candidate gene-based simple sequence repeat markers. Mol Breeding. 2014;34:1261–79.

[31] Babu BK, Dinesh P, Agrawal PK, Sood S, Chandrashekara C, Bhatt JC, et al Comparative genomics and association mapping approaches for blast resistant genes in finger millet using SSRs. PLoS ONE. 2014;9:e99182 10.1371/journal.pone.0099182 24915067 PMC4051690

[32] Ramakrishnan M, Ceasar S, Duraipandiyan V, Al-Dhabi N, Ignacimuthu S. Assessment of genetic diversity, population structure and relationships in Indian and non-Indian genotypes of finger millet (*Eleusine coracana* (L.) Gaertn) using genomic SSR markers. SpringerPlus. 2016:5:120 10.1186/s40064-015-1626-y 26900542 PMC4749518

[33] Federer W. Experimental Design: Theory and Application. Oxford & IBH Publishing Company 1967:1–591.

[34] Aru J, Okori P, Wanyera N. Identification of sources of blast resistance among drought tolerant finger millet accessions in Uganda. In African Crop Science Conference Proceedings. 2013;483–487.

[35] Wheeler BEJ. An Introduction of Plant Disease, John Wiley and Sons Limited, London 1969:301.

[36] Mackill DJ, Bonnman JM. Inheritance of blast resistance in near-isogenic lines of rice. Phytopathology 1992;82:746–9.

[37] Hammer Ø, Harper D, Ryan P. PAST: paleontological statistics software package for education and data analysis. Palaeontologia Electronica. 2001;4:9.

[38] Kole C, Dida MM, Devos KM. Cereals and Millets: Springer Berlin Heidelberg; 2006.

[39] Doyle JJ, Doyle JL. Isolation of plant DNA from fresh tissue. Focus. 1990;12:13–5.

[40] Ramakrishnan M, Ceasar SA, Duraipandiyan V, Al-Dhabi NA, Ignacimuthu S. Using molecular markers to assess the genetic diversity and population structure of finger millet (*Eleusine coracana* (L.) Gaertn.) from various geographical regions. Genet Resour Crop Evol. 2016;63:361–376.

[41] Pritchard JK, Stephens M, Donnelly P. Inference of population structure using multilocus genotype data. Genetics 2000;155:945–959. 10835412 10.1093/genetics/155.2.945 PMC1461096

[42] Karandikar R On the Markov Chain Monte Carlo (MCMC) method. Sadhana 2006;31:81–84.

[43] Evanno G, Regnaut S, Goudet J. Detecting the number of clusters of individuals using the software STRUCTURE: a simulation study. Molecular Ecology 2005;14:2611–2620. 15969739 10.1111/j.1365-294X.2005.02553.x

[44] Endelman JB, Jannink J-L. Shrinkage Estimation of the Realized Relationship Matrix. G3: Genes, Genomes, Genetics. 2012;2:1405–13. 10.1534/g3.112.004259 PMC3484671 23173092

[45] Bradbury P, Zhang Z, Kroon D, Casstevens T, Ramdoss Y, Buckler E. TASSEL: software for association mapping of complex traits in diverse samples. Bioinformatics. 2007;23:2633–5. 17586829 10.1093/bioinformatics/btm308

[46] Hao Z, Wang L, Huang F, Tao R. Expression patterns of defense genes in resistance of the panicles exserted from the caulis and from the tillers to neck blast in rice. Plant physiol and bioch. 2012;60:150–156. 10.1016/j.plaphy.2012.08.006 22940453

[47] Kurotani KI, Hattori T, Takeda S. Overexpression of a CYP94 family gene CYP94C2b increases internode length and plant height in rice. Plant Signal Behav. 2015;10:e1046667 10.1080/15592324.2015.1046667 26251886 PMC4623425

[48] Yeh SY, Chen HW, Ng CY, Lin CY, Tseng TH, et al Down-regulation of cytokinin oxidase 2 expression increases tiller number and improves rice yield. Rice (N Y). 2015;8:36. 10.1186/s12284-015-0070-5 PMC4671980 26643073

[49] Xu M, Zhu L, Shou H, Wu P. A PIN1 family gene, OsPIN1, involved in auxin-dependent adventitious root emergence and tillering in rice. Plant cell physiol. 2005;46:1674–1681. 16085936 10.1093/pcp/pci183

[50] Nakano T, Suzuki K, Fujimura T, Shinshi H. Genome-wide analysis of the ERF gene family in *Arabidopsis* and rice. Plant physiol. 2006;140:411–432. 16407444 10.1104/pp.105.073783 PMC1361313

[51] Tanaka W, Pautler M, Jackson D, Hirano H-Y. Grass meristems II: inflorescence architecture, flower development and meristem fate. Plant cell physiol. 2013;54:313–324. 10.1093/pcp/pct016 23378448

[52] Greco R, Stagi L, Colombo L, Angenent G, Sari-Gorla M, et al MADS box genes expressed in developing inflorescences of rice and sorghum. Mol Gen Genet. 1997;253:615–623. 9065695 10.1007/s004380050364

[53] Zhou Z, Sun L, Zhao Y, An L, Yan A, et al Zinc Finger Protein 6 (ZFP6) regulates trichome initiation by integrating gibberellin and cytokinin signaling in *Arabidopsis thaliana*. New Phytol. 2013;198:699–708. 10.1111/nph.12211 23506479

[54] Gamuyao R, Chin JH, Pariasca-Tanaka J, Pesaresi P, Catausan S, et al The protein kinase Pstol1 from traditional rice confers tolerance of phosphorus deficiency. Nature. 2012;488: 535–539. 10.1038/nature11346 22914168

[55] Markakis MN, De Cnodder T, Lewandowski M, Simon D, Boron A, et al Identification of genes involved in the ACC-mediated control of root cell elongation in *Arabidopsis thaliana*. BMC plant biology. 2012;12:208 10.1186/1471-2229-12-208 23134674 PMC3502322

[56] Xu P, Cai X-T, Wang Y, Xing L, Chen Q, et al HDG11 upregulates cell-wall-loosening protein genes to promote root elongation in *Arabidopsis*. J exp bot. 2014;65:4285–95. 10.1093/jxb/eru202 24821957 PMC4112634

[57] Zielinski RE. Calmodulin and calmodulin-binding proteins in plants. Annu Rev Plant Physiol Plant Mol Biol. 1998;49:697–725. 15012251 10.1146/annurev.arplant.49.1.697

[58] Anilkumar TB, Mantur SG, Madhukeshwara SS. Diseases of finger millet. Project coordination cell, all India coordinated samll millets improvent project, ICAR, GKVK, Bangalore 2003:1–126.

[59] Babu TK, Thakur RP, Upadhyaya HD, Reddy PN, Sharma R, Girish AG, et al Resistance to blast (*Magnaporthe grisea*) in a mini-core collection of finger millet germplasm. Eur J Plant Pathol. 2012;135:299–311.

[60] Takan JP, Chipili J, Muthumeenakshi S, Talbot NJ, Manyasa EO, Bandyopadhyay R, et al *Magnaporthe oryzae* populations adapted to finger millet and rice exhibit distinctive patterns of genetic diversity, sexuality and host interaction. Mol Biotechnol. 2012;50:145–58. 10.1007/s12033-011-9429-z 21701860

[61] Nagaraja A, Nanja Reddy Y, Anjaneya Reddy B, Patro T, Kumar B, Kumar J, et al Reaction of finger millet recombinant inbred lines (RILs) to blast. Crop Research (Hissar) 2010;39:120–2.

[62] Wu JL, Sinha PK, Variar M, Zheng KL, Leach JE, Courtois B, et al Association between molecular markers and blast resistance in an advanced backcross population of rice. Theor Appl Genet. 2004;108:1024–32. 15067388 10.1007/s00122-003-1528-1

[63] Esele JP. Diseases of Finger Millet—A Global Overview. Sorghum and Millets Diseases: Iowa State Press; 2008 p. 19–26.

[64] Koutroubas SD, Katsantonis D, Ntanos DA, Lupotto E. Blast disease influence on agronomic and quality traits of rice varieties under Mediterranean conditions. Turk J Agric For. 2009;33:487–94.

[65] McRae W. Report of the Imperial Mycologist. Agric Res Inst Pusa Scientific Reports. 1922:1921–22,44–50.

[66] Venkatarayan S. Disease of ragi (*Eleusine coracana*). Mysore Agric J. 1947;24:50–7.

[67] Upadhyaya H, Gowda CLL, Pundir RPS, Reddy VG, Singh S. Development of Core Subset of Finger Millet Germplasm Using Geographical Origin and Data on 14 Quantitative Traits. Genet Resour Crop Evol. 2006;53:679–85.

[68] Babu BK, Agrawal P, Pandey D, Sood S, Chandrashekara C, Kumar A. Molecular analysis of world collection of finger millet accessions for blast disease resistance using functional SSR markers. SABRAO Journal of Breeding and Genetics. 2014;46:202–16.

[69] Joel A, Kumaravadivel N, Nirmalakumari A, Senthil N, Mohanasundaram K, Raveendran T, et al A high yielding Finger millet variety CO(Ra) 14. Madras Agric J. 2005;92:375–80.

[70] Abdurakhmonov I, Abdukarimov A. Application of association mapping to understanding the genetic diversity of plant germplasm resources. Int J Plant Genomics 2008;2008:1–18. 10.1155/2008/574927 PMC2423417 18551188

[71] Singh BD, Singh AK. Marker-Assisted Plant Breeding: Principles and Practices. Springer, New Delhi 2015:217–56.

[72] Vignal A, Milan D, Sancristobal M, Eggen A. A review on SNP and other types of molecular markers and their use in animal genetics. Genet Sel Evol. 2002;34:275–305. 12081799 10.1186/1297-9686-34-3-275 PMC2705447

[73] Ashkani S, Rafii MY, Rahim HA, Latif MA. Mapping of the quantitative trait locus (QTL) conferring partial resistance to rice leaf blast disease. Biotechnol Lett. 2013;35:799–810. 10.1007/s10529-012-1130-1 23315158

[74] De Villiers S, Michael V, Manyasa E, Saiyiorri A, Deshpande S. Compilation of an informative microsatellite set for genetic characterization of East African finger millet (*Eleusine coracana*). Electronic Journal of Biotechnology. 2015;18:77–82.

[75] Bennetzen JL, Freeling M. The unified grass genome: synergy in synteny. Genome Res. 1997;7:301–306 9110169 10.1101/gr.7.4.301

[76] Nirgude M, Babu BK, Shambhavi Y, Singh UM, Upadhyaya HD, Kumar A. Development and molecular characterization of genic molecular markers for grain protein and calcium content in finger millet (*Eleusine coracana* (L.) Gaertn.). Mol Biol Rep. 2014;41:1189–200. 10.1007/s11033-013-2825-7 24477581

[77] Bennetzen JL, Schmutz J, Wang H, Percifield R, Hawkins J, Pontaroli AC, et al Reference genome sequence of the model plant *Setaria*. Nat Biotech. 2012;30:555–61. 10.1038/nbt.2196 22580951

[78] Zhang G, Liu X, Quan Z, Cheng S, Xu X, Pan S, et al Genome sequence of foxtail millet (*Setaria italica*) provides insights into grass evolution and biofuel potential. Nat Biotech. 2012;30:549–54. 10.1038/nbt.2195 22580950

[79] Muthamilarasan M, Prasad M. Advances in *Setaria* genomics for genetic improvement of cereals and bioenergy grasses. Theor Appl Genet. 2015;128:1–14. 10.1007/s00122-014-2399-3 25239219

[80] Sock J, Rohringer R, Kang Z. Extracellular β-1, 3-Glucanases in stem rust-affected and abiotically stressed wheat leaves immunocytochemical localization of the enzyme and detection of multiple forms in gels by activity staining with dye-labeled laminarin. Plant physiology. 1990;94:1376–1389. 16667843 10.1104/pp.94.3.1376 PMC1077388

[81] Opiyo SO, Moriyama EN. Mining Cytochrome b561 Proteins from Plant Genomes. Int. J. Bioinformatics Research and Applications. 2010;6:209–21. 10.1504/IJBRA.2010.032122 PMC2846449 20223741

[82] Ashikari M. Cytokinin oxidase regulates rice grain production. Science. 2005;309:741–745. 15976269 10.1126/science.1113373

[83] Kumar A, Mirza N, Charan T, Sharma N, Gaur VS. Isolation, characterization and immunolocalization of a seed dominant CaM from finger millet (*Eleusine coracana* L. Gartn.) for studying its functional role in differential accumulation of calcium in developing grains. Appl Biochem Biotechnol. 2014;172:2955–73. 10.1007/s12010-013-0714-0 24469585

[84] FAO. Sorghum and millets in human nutrition FAO food and nutrition series, No 27 Food and agriculture organization, Rome, Italy 1995:186.

[85] Singh UM, Pandey D, Kumar A. Determination of calcium responsiveness towards exogenous application in two genotypes of *Eleusine coracana* L. differing in their grain calcium content. Acta Physiol Plant. 2014;36:2521–2529.

