# Supplementary material for: Tracing QTLs for Leaf Blast Resistance and Agronomic Performance of Finger Millet (Eleusine coracana (L.) Gaertn.) Genotypes through Association Mapping and in silico Comparative Genomics Analyses
Source: PLoS One. 2016 Jul 14;11(7):e0159264. doi: 10.1371/journal.pone.0159264 (PMC4944987; doi:10.1371/journal.pone.0159264)

Figure A. Design layout of the experiment.

Design: Augmented

Test entries: 122   Checks: 6 (Resistant 3, Susceptible 3)

Number of blocks: 14   Number of test entries in each block: 9

Replications per check: 24   Block dimension: 9.5 x 3 m   Spacing: 20 x 10 cm

Checks in two rows and Test genotypes grown in three rows

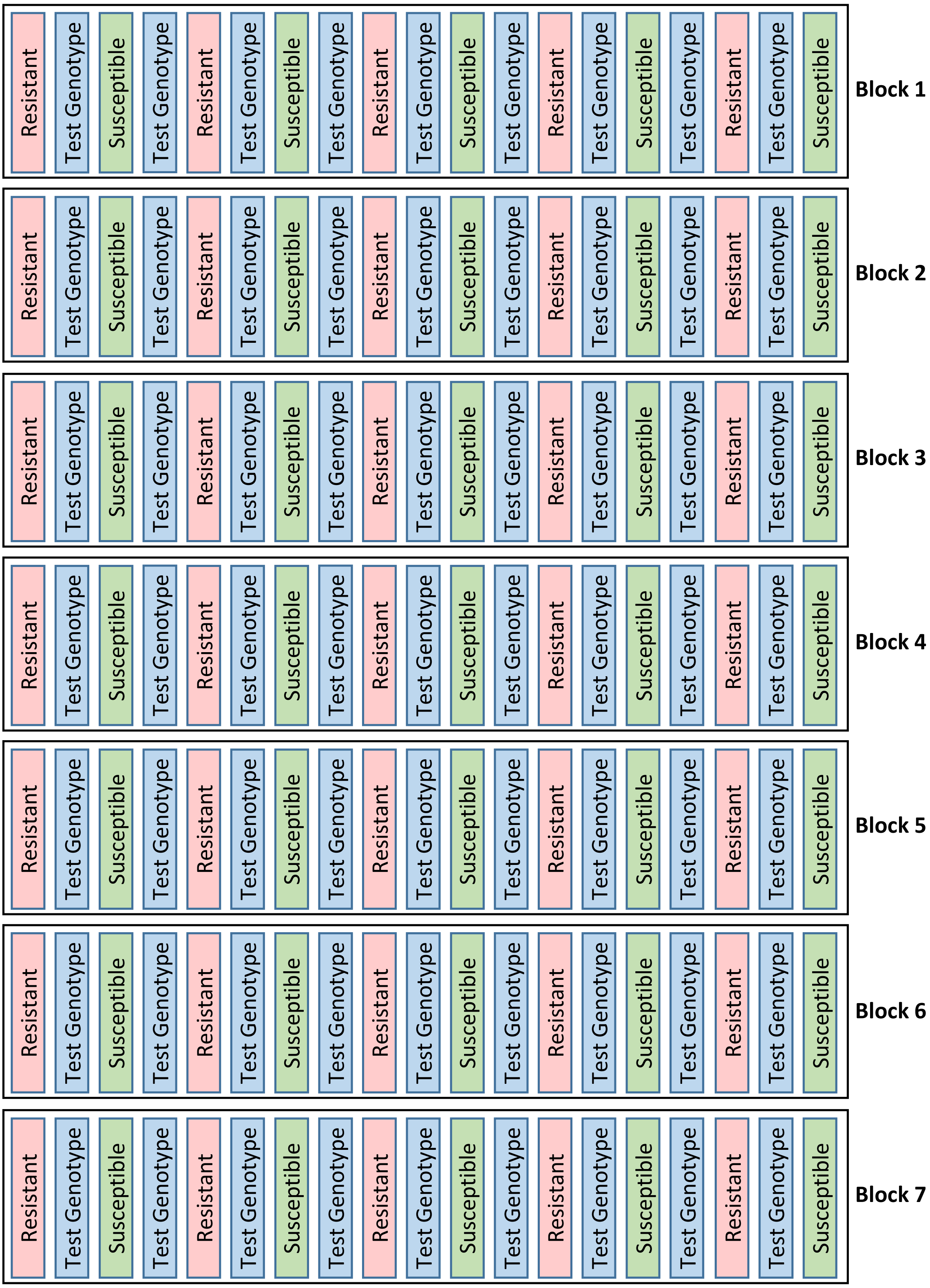

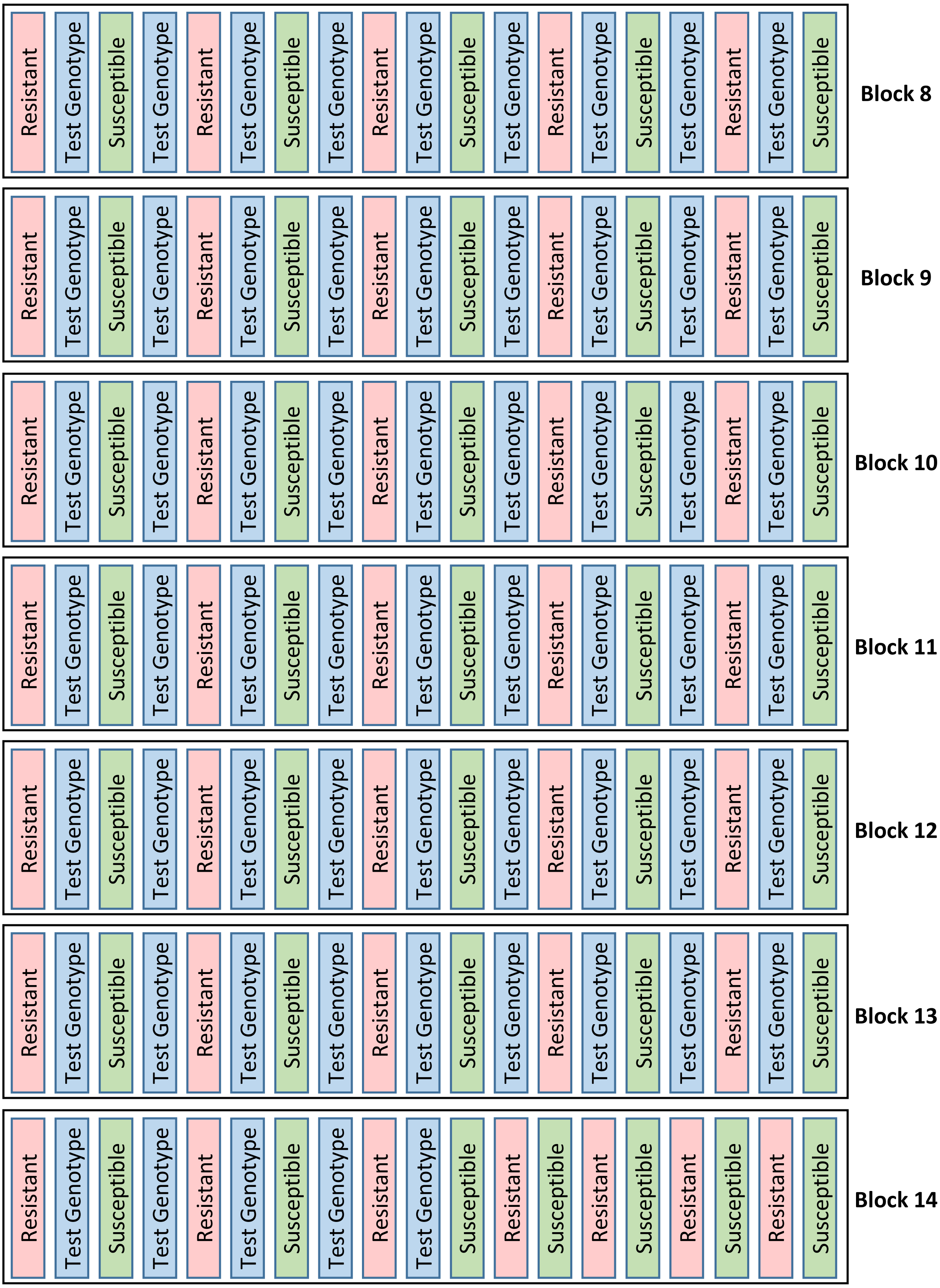
Resistant Test Genotype Susceptible Test Genotype Resistant Test Genotype Susceptible Test Genotype Resistant Test Genotype Susceptible Test Genotype Resistant Test Genotype Susceptible Test Genotype Resistant Test Genotype Susceptible
Block 8
Resistant Test Genotype Susceptible Test Genotype Resistant Test Genotype Susceptible Test Genotype Resistant Test Genotype Susceptible Test Genotype Resistant Test Genotype Susceptible Test Genotype Resistant Test Genotype Susceptible
Block 9
Resistant Test Genotype Susceptible Test Genotype Resistant Test Genotype Susceptible Test Genotype Resistant Test Genotype Susceptible Test Genotype Resistant Test Genotype Susceptible Test Genotype Resistant Test Genotype Susceptible
Block 10
Resistant Test Genotype Susceptible Test Genotype Resistant Test Genotype Susceptible Test Genotype Resistant Test Genotype Susceptible Test Genotype Resistant Test Genotype Susceptible Test Genotype Resistant Test Genotype Susceptible
Block 11
Resistant Test Genotype Susceptible Test Genotype Resistant Test Genotype Susceptible Test Genotype Resistant Test Genotype Susceptible Test Genotype Resistant Test Genotype Susceptible Test Genotype Resistant Test Genotype Susceptible
Block 12
Resistant Test Genotype Susceptible Test Genotype Resistant Test Genotype Susceptible Test Genotype Resistant Test Genotype Susceptible Test Genotype Resistant Test Genotype Susceptible Test Genotype Resistant Test Genotype Susceptible
Block 13
Resistant Test Genotype Susceptible Test Genotype Resistant Test Genotype Susceptible Test Genotype Resistant Test Genotype Susceptible Resistant Susceptible Resistant Susceptible Resistant Susceptible Resistant Susceptible
Block 14

Figure B. Screen shot image of comparative genomics analysis with QTL UGEP50 in genome of foxtail millet. QTL UGEP50 was associated with candidate gene Cytochrome P450 CYP2 at 74.112 kb distances in foxtail millet Scaffold-3 for internode length and plant growth

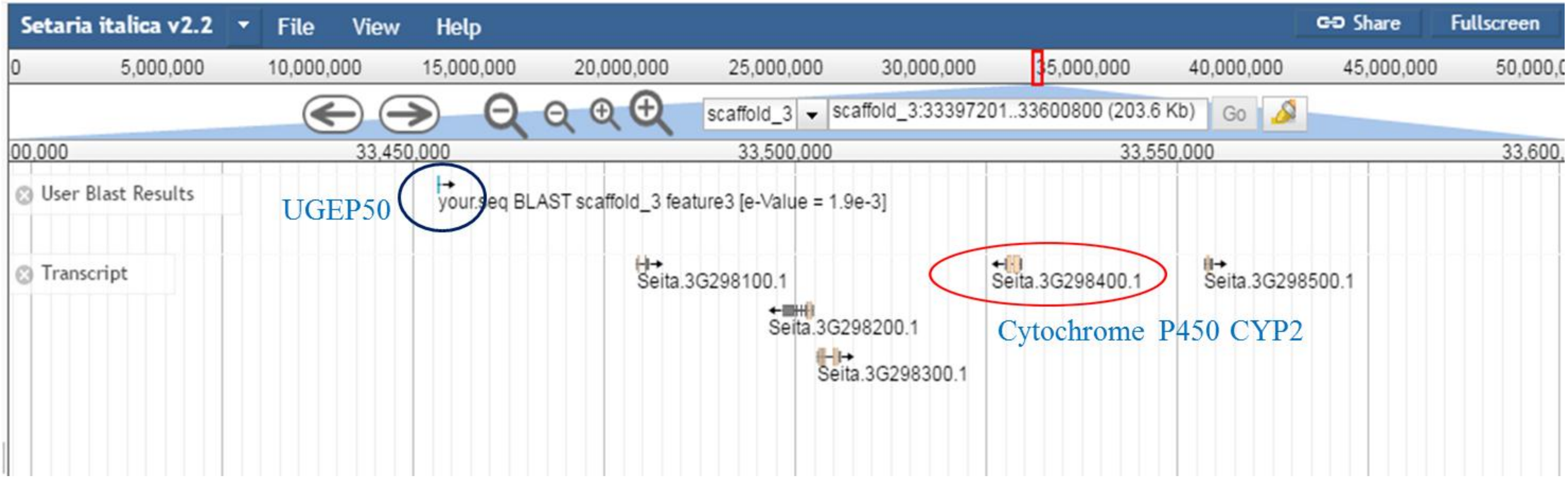

Figure C. Screen shot image of comparative genomics analysis with QTL UGEP57 in genome of rice. QTL UGEP57 was associated with candidate gene PMEI at 13.201 kb distance in rice chromosome 4 for primary root growth

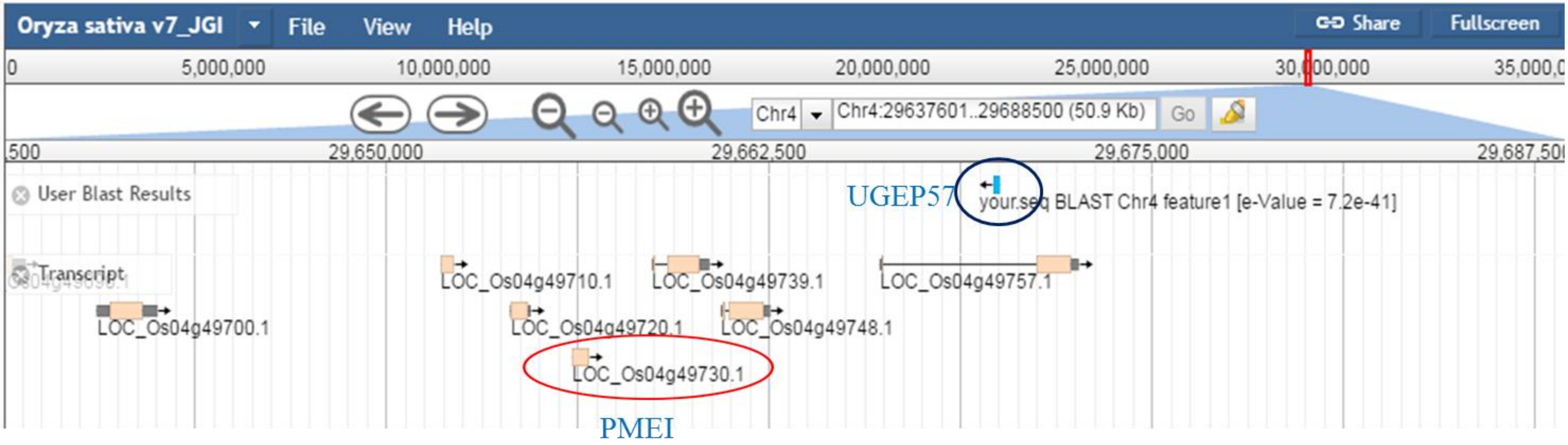

Figure D. Screen shot image of comparative genomics analysis with QTL UGEP19 in genome of rice. QTL UGEP19 was associated with candidate gene CaM-binding protein at 61.899 kb distance in rice chromosome 10 for calcium accumulation in finger millet grains

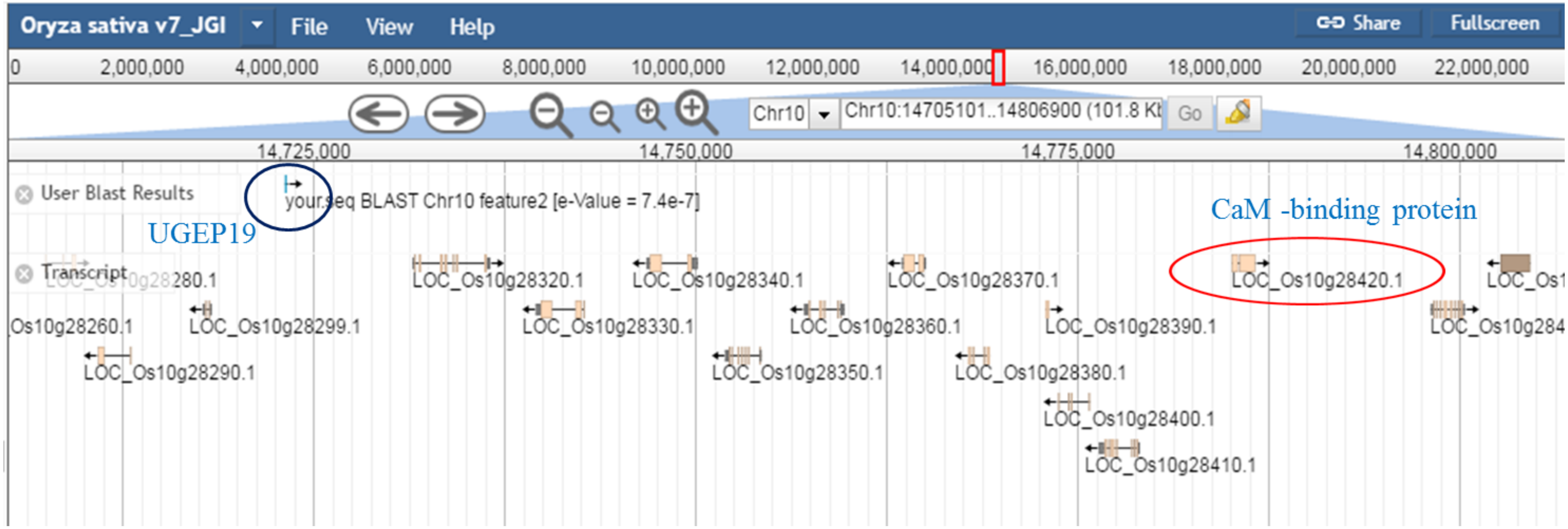

Figure E. Screen shot image of comparative genomics analysis with QTL UGEP98 in genome of hall's panicgrass. QTL UGEP98 was associated with candidate gene ARF at 3.491 kb distances in hall's panicgrass chromosome 6 for tiller growth and development

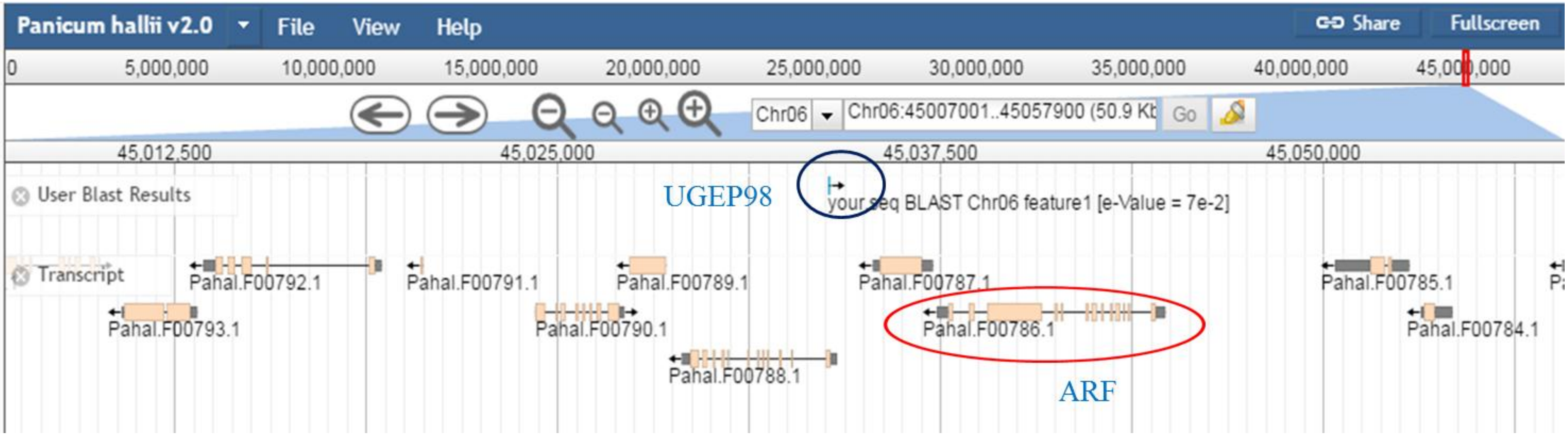

Figure F. Screen shot image of comparative genomics analysis with QTL UGEP104 in genome of *Brachypodium distachyon*. QTL UGEP104 was associated with candidate gene ERF at 58.488 kb distances in *Brachypodium distachyon* chromosome 1 for Flower development

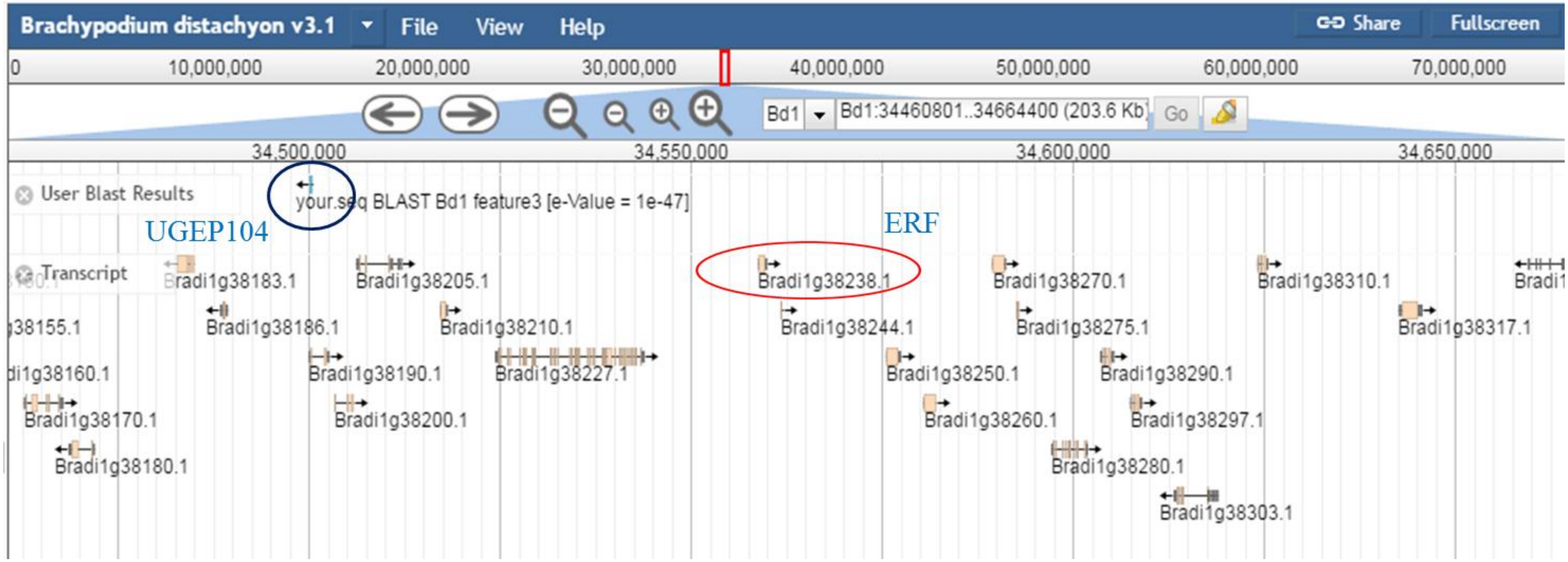

Figure G. Screen shot image of comparative genomics analysis with QTL UGEP104 in genome of *Brachypodium distachyon*. QTL UGEP104 was associated with candidate gene MADS transcription factor at 1.256 kb distances in *Brachypodium distachyon* chromosome 5 for Meristem determinacy and development

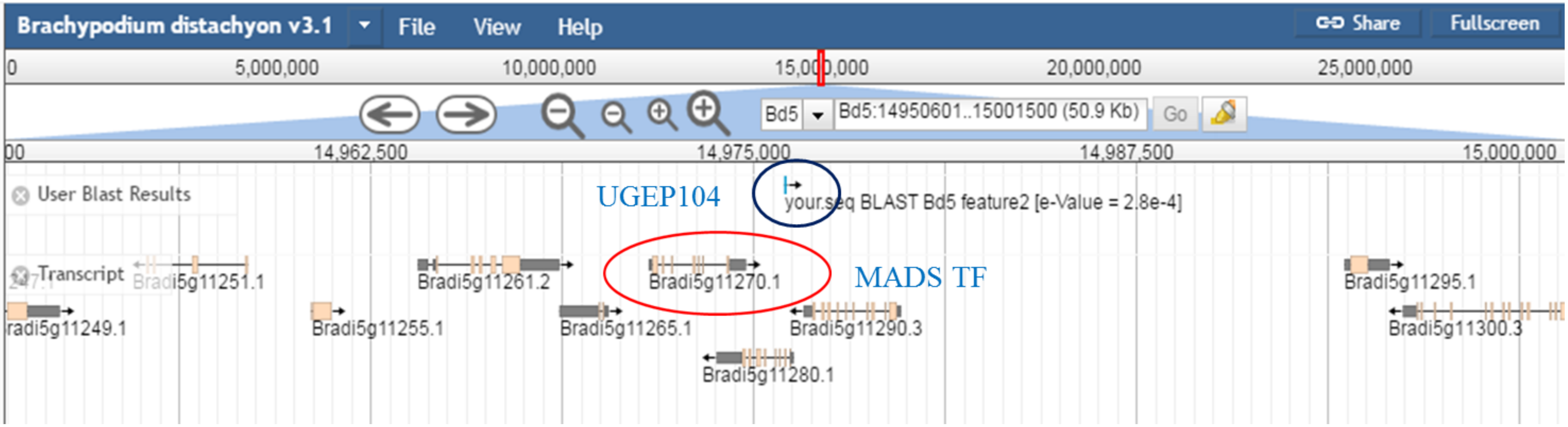

Figure H. Screen shot image of comparative genomics analysis with QTL UGEP104 in genome of switchgrass. QTL UGEP104 was associated with candidate gene MADS box protein at 82.637 kb distances in switchgrass chromosome 3a for Inflorescence development

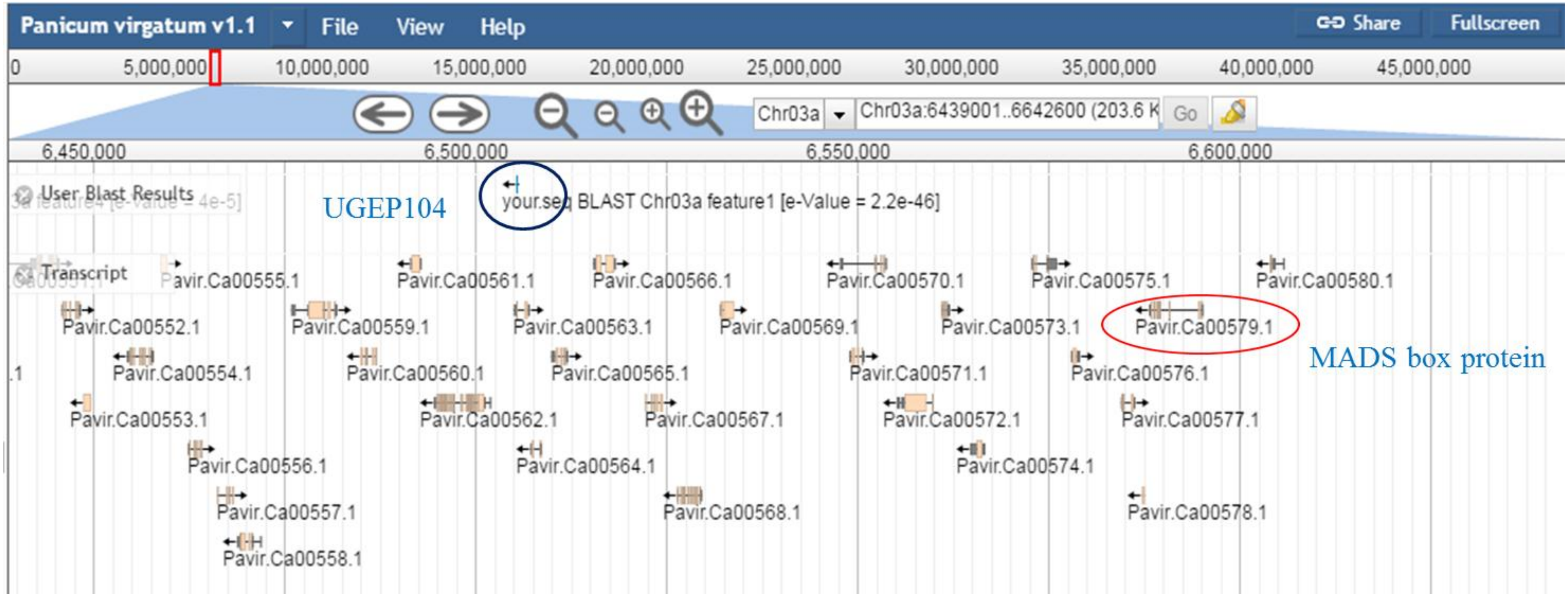

Figure I. Screen shot image of comparative genomics analysis with QTL UGEP104 in genome of foxtail millet. QTL UGEP104 was associated with candidate gene ZF-C2H2_6 at 26.657 kb distances in foxtail millet Scaffold_7 for Trichome development on the inflorescence

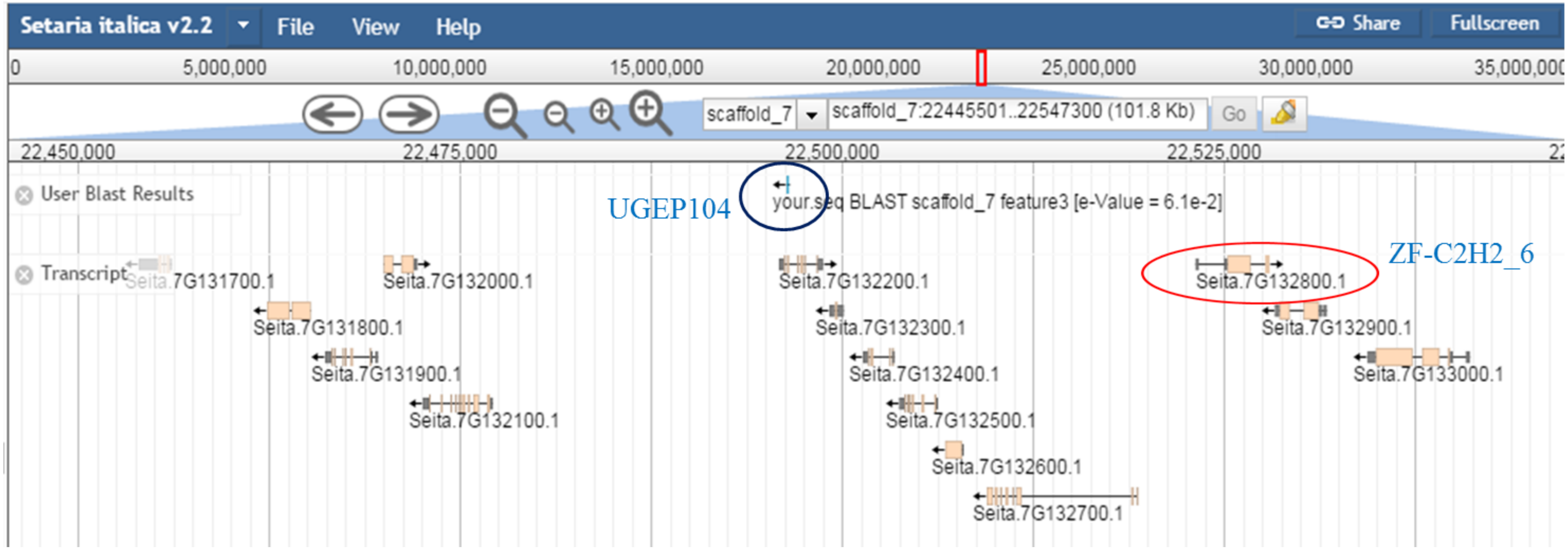

Figure J. Screen shot image of comparative genomics analysis with QTL UGEP9 in genome of foxtail millet. QTL UGEP9 was associated with candidate gene Ser/Thr protein kinase at 6.292 kb distances in foxtail millet Scaffold_4 for early root growth and development

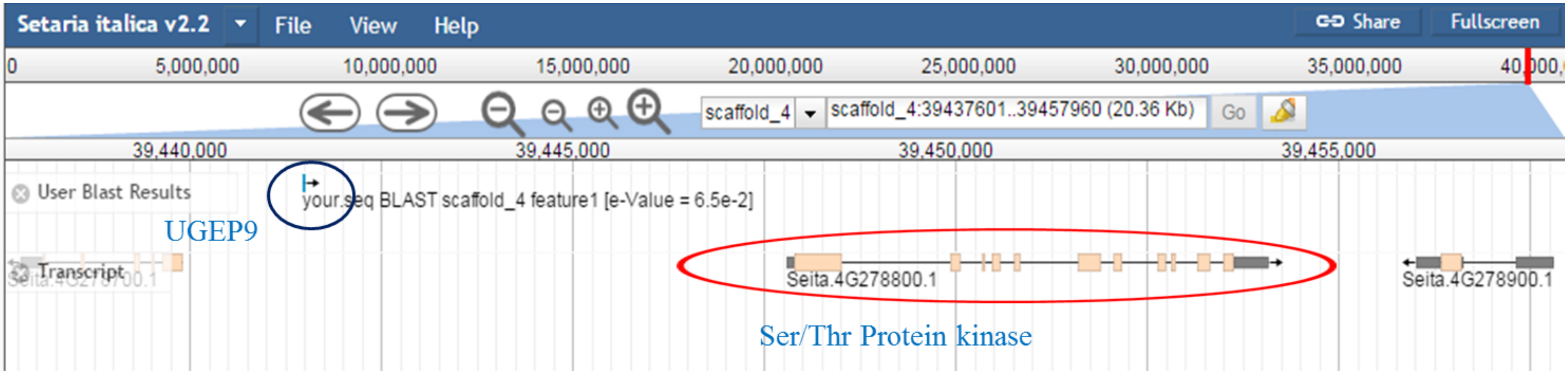

Figure K. Screen shot image of comparative genomics analysis with QTL UGEP9 in genome of *Brachypodium stacei*. QTL UGEP9 was associated with candidate gene Cytochrome P450 CYP2 at 51.917 kb distances in *Brachypodium stacei* chromosome 5 for Primary root growth and development

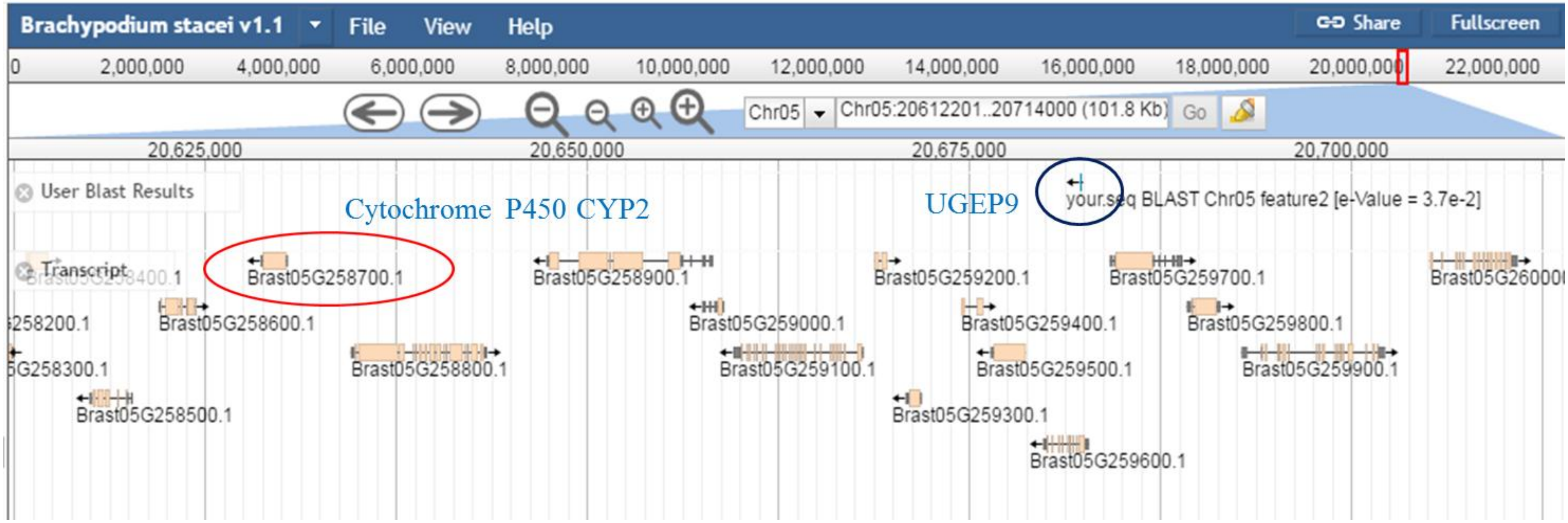

Supplement: S1 File — Figures A-K, (A) The design layout of the experiment. The test genotypes were grown alternated with resistant and susceptible checks to provide adequate disease load throughout cropping season; (B) Screen shot image of comparative genomics analysis with QTL UGEP50 in genome of foxtail millet. QTL UGEP50 was associated with candidate gene Cytochrome P450 CYP2 at 74.112 kb distances in foxtail millet Scaffold-3 for internode length and plant growth; (C) Screen shot image of comparative genomics analysis with QTL UGEP57 in genome of rice. QTL UGEP57 was associated with candidate gene PMEI at 13.201 kb distance in rice chromosome 4 for primary root growth; (D) Screen shot image of comparative genomics analysis with QTL UGEP19 in genome of rice. QTL UGEP19 was associated with candidate gene CaM-binding protein at 61.899 kb distance in rice chromosome 10 for calcium accumulation in finger millet grains; (E) Screen shot image of comparative genomics analysis with QTL UGEP98 in genome of hall’s panicgrass. QTL UGEP98 was associated with candidate gene ARF at 3.491 kb distances in hall’s panicgrass chromosome 6 for tiller growth and development; (F) Screen shot image of comparative genomics analysis with QTL UGEP104 in genome of Brachypodium distachyon. QTL UGEP104 was associated with candidate gene ERF at 58.488 kb distances in Brachypodium distachyon chromosome 1 for Flower development; (G) Screen shot image of comparative genomics analysis with QTL UGEP104 in genome of Brachypodium distachyon. QTL UGEP104 was associated with candidate gene MADS transcription factor at 1.256 kb distances in Brachypodium distachyon chromosome 5 for Meristem determinacy and development; (H) Screen shot image of comparative genomics analysis with QTL UGEP104 in genome of switchgrass. QTL UGEP104 was associated with candidate gene MADS box protein at 82.637 kb distances in switchgrass chromosome 3a for Inflorescence development; (I) Screen shot image of comparative genomics analysis with QTL U [file pone.0159264.s001.pdf]
