# Supplementary material for: Tracing QTLs for Leaf Blast Resistance and Agronomic Performance of Finger Millet (Eleusine coracana (L.) Gaertn.) Genotypes through Association Mapping and in silico Comparative Genomics Analyses
Source: PLoS One. 2016 Jul 14;11(7):e0159264. doi: 10.1371/journal.pone.0159264 (PMC4944987; doi:10.1371/journal.pone.0159264)
Supplement: S2 File — Tables A-B, (A) Agronomic performance and percentage of disease incidences (PDI) of the 128 finger millet genotypes; the data were collected after 45 and 90 days of sowing the seeds for PDI and agronomic traits respectively; (B) List of 87 genomic SSR primers with primer sequence (5’ to 3’), SSR motif and size of markers (bp) used for the analysis of population structure of 128 finger millet genotypes. (PDF) [file pone.0159264.s002.pdf]

Table A. List of 87 genomic SSR primers with primer sequence (5' to 3'), SSR motif and size of markers (bp) used for the analysis of population structure of 128 finger millet genotypes

Name of the primer	Primer sequence (5' to 3')	SSR motif	Size of markers (bp)
SSR-01	(F) GCGAAAACACAATGCAAAAA<br>(R) GCGTTGGTTGGACCTGAC	$(AC)_{19}(AT)_{29}$	400
SSR-02	(F) TCCTCCCTCCCTTCGCCCACTG<br>(R) CGATGTTCGCCATGGCAGCGACC	$(GA)_{26}$	1000
SSR-06	(F) GCCTCGAGCATCATCATCAG<br>(R) ATCAACCTGCACTTGCCTGG	$(TC)_{22}TT(GT)_{19}$	700
SSR-08	(F) TTCCCTGTTAAGAGAGAAATC<br>(R) GTGTATTTGGTGAAAGCAAC	$(GA)_{17}AA(GA)_{19}$	600
SSR-10	(F) CTTTGTCTATCTCAAGACAC<br>(R) TTGCAGATGTTCTTCCTGATG	$(GA)_{14}$	500
UGEP1	(F) TTCAGTGGTGACGGAAGTTCT<br>(R) GGCTCCATGAAGAGCTTGAC	$(TC)_{11}$	233
UGEP3	(F) CCACGAGGCCATACTGAATAG<br>(R) GATGGCCACTAGGGATGTTG	$(CA)_{7}N_{12}(GA)_{15}$	206
UGEP5	(F) TGTACACAACACCACACTGATG<br>(R) TTGTTTGGACGTTGGATGTG	$(TC)_{12}AC(TC)_{4}$	215
UGEP6	(F) AGCTGCAGTTTCAGTGGATTC<br>(R) TCAACAAGGTGAAGCAGAGC	$(GA)_{3}TA(GA)_{9}$	229
UGEP8	(F) ATTTCCGCCATCACTCCAC<br>(R) AGACGCAAATGGGTAAATGTC	$(GA)_{13}$	297
UGEP10	(F) AAACGCGATGAATTTTAAGCTC<br>(R) CTATGTCGTGTCCCATGTCG	$(GA)_{19}$	400
UGEP11	(F) CCTCGAGTGGGGATCCAG<br>(R) AAGACGCTGGTGGAAATAGC	$(CT)_{12}$	153
UGEP12	(F) ATCCCCACCTACGAGATGC<br>(R) TCAAAGTGATGCGTCAGGTC	$(CT)_{22}$	230
UGEP15	(F) AAGGCAATCTCGAATGCAAC<br>(R) AAGCCATGGATCCTTCCTTC	$(CT_{)22}$	180
UGEP18	(F) TTGCATGTGTTGCTTTTTGC<br>(R) TGTTCTTGATTGCAAACTGATG	$(CT)_{12}$	318
UGEP21	(F) CAATTGATGTCATTGGGACAAC<br>(R) GTATCCACCTGCATGCCAAC	$(GA)_{16}$	225
UGEP24	(F) GCCTTTTGATTGTTCAACTCG<br>(R) CGTGATCCCTCTCCTCTCTG	$(GA_{)26}$	183

Name of the primer	Primer sequence (5' to 3')	SSR motif	Size of markers (bp)
UGEP26	(F) ATGGGGTTAGGGTTCGAGTC<br>(R) TGTCCCTCACTCGTCTCCTC	$(CGG)_7$	227
UGEP31	(F) ATGTTGATAGCCGGAAATGG<br>(R) CCGTGAGCCTCGAGTTTTAG	$(GA)_{12}$	241
UGEP52	(F) TCATGCTAGCTTCAACACAACC<br>(R) TGCTGGGTGAAACCCTAGAC	$(GA)_{16}$	215
UGEP53	(F) TGCCACAACTGTCAACAAAAG<br>(R) CCTCGATGGCCATTATCAAG	$(AG)_{26}$	226
UGEP56	(F) CTCCGATACAGGCGTAAAGG<br>(R) ACCATAATAGGGCCGCTTG	$(GT)_{12}$	162
UGEP60	(F) AGCTCTGCTTGGTGGAGAAG<br>(R) TTTTCTACTGGTGGGCGAAG	$(GA)_{37}$	240
UGEP65	(F) AGTGCTAGCTTCCCATCAGC<br>(R) ACCGAAACCCTTGTCAGTTC	$(CT)_{19}$	226
UGEP68	(F) CGGTCAGCATATAACGAATGG<br>(R) TCATTGATGAATCCGACGTG	$(CT)_{14}$	232
UGEP76	(F) GCACGTACGGATTCACATTG<br>(R) GGTACGGAGACATCGACACC	$(CAG)_7$	168
UGEP77	(F) TTCGCGCGAAATATAGGC<br>(R) CTCGTAAGCACCCACCTTTC	$(CT)_{19}$	245
UGEP78	(F) AAGCAATCAACAAAGCCTTTTC<br>(R) TACAACGTCCAGGCAACAAG	$(GA)_{14}$	244
UGEP81	(F) AAGGGCCATACCAACACTCC<br>(R) CACTCGAGAACCGACCTTTG	$(GT)_{12}$	192
UGEP90	(F) GGCCTTTGCAGTCATGTGAG<br>(R) CGACTCCAGGTGTTGTTGG	$(CT)_{11}/(CT)_8$	232
UGEP102	(F) ATGCAGCCTTTGTCATCTCC<br>(R) GATGCCTTCCTTCCCTTCTC	$(TG)_{17}$	184
UGEP104	(F) TCAGCACCACCTGAATAGG<br>(R) AATAGGGAGGGCGAAGACTC	$(CT)_{11}$	189
UGEP106	(F) AATTCCATTCTCTCGCATCG<br>(R) TGCTGTGCTCCTCTGTTGAC	$(AC)_{12}$	175
UGEP107	(F) TCATGCTCCATGAAGAGTGTG<br>(R) TGTCAAAAACCGGATCCAAG	$(GA)_{15}$	224
UGEP108	(F) GTTGGCTGCTCTGCTTATCC<br>(R) TATCTGCTTGTGCAGCTTCG	$(CTG)_6(CAG)_2$	150
UGEP110	(F) AAATTCGCATCCTTGCTGAC<br>(R) TGACAAGAGCACACCGACTC	$(CT)_{12}$	192

Name of the primer	Primer sequence (5’ to 3’)	SSR motif	Size of markers (bp)
UGEP7	(F) GCCAACAGTTCGCTCTGC<br>(R) CTGTCTCCCGTTCTCTCCTG	$(GA)_{14}$	500
UGEP9	(F) GGTGGAGAAGGGAGAAGGAG<br>(R) TTGAAGGCCCAATAGCTACG	$(GGA)_{7}$	239
UGEP13	(F) AGCACGATCGAAATCCATTC<br>(R) AGATCTCGCAGCGGTTTATG	$(TC)_{18}$	206
UGEP16	(F) TCTCCAGCTACATATGTTTTACT<br>(R) ATATATGGGACAACCATCCA	$(CT)_{14}(AT)_{16}$	197
UGEP17	(F) CATTGGTGTCGTTCACTTGG<br>(R) TGCACCGTATGAGCAACAAG	$(GTT)_{7}$	218
UGEP19	(F) GGCAAGGATACCTTCATTCG<br>(R) TACCTCTGCTCTGCACATGG	$(GA)_{18}$	230
UGEP20	(F) GGGGAAGGCAATGATATGTG<br>(R) TTGGGGAGTGCCAACAATAC	$(GA)_{20}$	153
UGEP22	(F) ACAGAGACACGGTGGAGGTG<br>(R) CACCATGTCATCGTCACCAG	$(TC)_{29}$	227
UGEP25	(F) GTTCGGCCTCATCGTCAG<br>(R) TTTATCCGACGTGACAGGTG	$G_{8}(GA)_{19}$	292
UGEP27	(F) TTGCTCTGAGGTTGTGTTGC<br>(R) TCAAGCATAGTGCCCTCCTC	$(GA)_{19}$	247
UGEP28	(F) GAAGTTGCCATCCATGCTTC<br>(R) AGGAACCGCCAGGAAATTAG	$(TC)_{17}$	228
UGEP29	(F) TACCATTCCATTGCATCCAC<br>(R) TTAATTAGCACTTGGGCATGG	$(CT)_{12}$	234
UGEP33	(F) TAGCCCGTTTGCTTGTTTTG<br>(R) AAGGCCCTAGAACGTCAAGC	$(TC)_{18}$	216
UGEP34	(F) AAGATCCTCACACGCAAACAC<br>(R) ACCCTCAACTGGTGCCATAG	$(CA)_{12}$	196
UGEP45	(F) GGTCTCTTCAAAGCCACTGC<br>(R) AACCAACCAATCCCAATATCC	$(GA)_{13}$	216
UGEP46	(F) CAAGTCAAAACATTCAGATGG<br>(R) CCACTCCATTGTAGCGAAAC	$(GA)_{14}$	163
UGEP47	(F) ACAGGCCCCATAAATTCCAC<br>(R) CCCCAATCATTCTTGTATGC	$(GA)_{18}$	198
UGEP50	(F) CACCCAGACATGACCCTTTC<br>(R) ACTAGTGCAGGGTGGTCTCG	$(CAA)_{7}$	249
UGEP51	(F) CAGCAGCACGACTGACCTG<br>(R) TGCCACCTGACTACTCTCTGC	$(GT)_{11}$	299

Name of the primer	Primer sequence (5’ to 3’)	SSR motif	Size of markers (bp)
UGEP54	(F) ACATTCCTGTTCGGGTTCTG<br>(R) TCGTGCACCATACCAATAGC	$(GTT)_8$	222
UGEP57	(F) CCATGGGTTCATCAAACACC<br>(R) ACATGAGCTCGCGTATTGC	$(AG)_{16}$	445
UGEP58	(F) CATGGTGGTGGAGAGAAACC<br>(R) GCAATGGTGACATTGAGCTG	$(CA)_{15}$	194
UGEP59	(F) ACGTGCTACGAGTGCTTGTG<br>(R) AAACCCTAAGCCCCTCAATC	$(GA)_{11}$	248
UGEP62	(F) GTGTGACGTGGCATACTTGG<br>(R) CACGCGAGGTAGGGTAGAAG	$(TC)_{13}$	194
UGEP64	(F) GTCACGTCGATTGGAGTGTG<br>(R) TCTCACGTGCATTTAGTCATTG	$(CT)_{23}$	206
UGEP66	(F) CAGATCTGGGTAGGGCTGTC<br>(R) GATGGTGGTTCATGCCAAC	$(AG)_{29}$	219
UGEP67	(F) CTCCTGATGCAAGCAAGGAC<br>(R) AGGTGCCGTAGTTTGTGCTC	$(TC)_{22}TT(GT)_5$	229
UGEP69	(F) GCATGAGCGGGAGAATAAAG<br>(R) AGCTAGCCAGAAGGCATGAG	$(GT)_9AT(GT)_7$	171
UGEP70	(F) TGTTGTCGCTGGACTCGTAG<br>(R) AACCACACCCGGTTCCTC	$(TCC)_7$	235
UGEP73	(F) GGTCAAAGAGCTGGCTATCG<br>(R) ACCAGAACCGAATCATGAGG	$(CT)_4CC(CT)_{10}$	229
UGEP74	(F) AGATCTGGCACCCATTTCC<br>(R) AGTCATCGGGGAGGGAAG	$(CT)_{21}$	223
UGEP75	(F) GCATTGGACGAAATAAATTGG<br>(R) GCAAATGTGCATGGTAGCAC	$(AC)_{19}(AT)_{29}$	400
UGEP79	(F) CCACTTTGCCGCTTGATTAG<br>(R) TGACATGAGAAGTGCCTTGC	$(CT)_{12}$	173
UGEP80	(F) TCCTGATCATCCTCATCATCC<br>(R) CCACTGTTTGAGCATGTTGG	$(CAT)_7$	185
UGEP83	(F) TAAGCTACGGCAGCGTCAG<br>(R) TCTACGCGTACGTGACCATC	$(GA)_{26}$	194
UGEP84	(F) GGAACTTCCGTCAGTCCTTG<br>(R) TGGGGAAGGTGTTGAATCTC	$(CT)_{24}$	195
UGEP86	(F) ACTCCCCTCACTCCCACAC<br>(R) CAGCCACCCTTGGACATC	$(CCT)_8$	222
UGEP87	(F) CTTCCCCGGACAAAGTGAAC<br>(R) AAGAGACAAGGCAGGGGAAG	$(TC)_{39}$	152

Name of the primer	Primer sequence (5’ to 3’)	SSR motif	Size of markers (bp)
UGEP88	(F) ACCTTGATCAGGGGTGCTC<br>(R) TTTTGAGTTTGCTCCCATGAC	$(CT)_{20}CC(CT)_{8}$	227
UGEP91	(F) GTGGCGAGAGAGGATGATTC<br>(R) TTCTCTAGCACGGCTTCACC	$(AC)_{8}(AG)_{14}$	236
UGEP93	(F) TGGCCTCGTTAGGTGAAGTC<br>(R) AGCACCAAAACTCCCACAAC	$(TC)_{21}$	174
UGEP95	(F) AGGGGACGCTTGGTTATTTG<br>(R) GCCTCTACCTGTCTCC GTTG	$(TC)_{14}$	208
UGEP96	(F) TAATGGGCCTAATGGCAATG<br>(R) CAAAATCCGAGCCAAGATTC	$(CT)_{10}$	211
UGEP97	(F) TTGAGCAAGGCTCCTCCTAC<br>(R) TCAACTCCTCAACCCTTTGG	$(GT)_{8}TT(GT)_{13}$	231
UGEP98	(F) GTCTTCCATTTGCAGCAACC<br>(R) ACGCGTACTGACGTGCTTG	$(GCC)_{8}$	223
UGEP100	(F) TCAAGTGTTTCTGGCACTCG<br>(R) TGCAAAAGCTGGGAGTAAC	$(CT)_{12}$	223
UGEP101	(F) GCTCACTTACCCATGGCTTC<br>(R) GAAATGTGGGGCACATAAGG	$(GA)_{7}AA(GA)_{19}$	234
UGEP103	(F) AGGGATGTCTTCAGCATTGG<br>(R) ACCGTGCACCATATCAACAG	$(GTT)_{8}$	239
UGEP105	(F) CTCCCGCTGTCCCATATTAC<br>(R) TTTCTCCAGCCACAAAGGAC	$(GA)_{20}$	161
UGEP109	(F) ACGCGACTTTGACCTGCTAC<br>(R) AAAAGGACGGGAAAGGAGAG	$(GA)_{13}$	208
UGEP111	(F) CTCTCCCTGAGTTGGTCTGC<br>(R) TTGGTCAGAAGCATCAGGTG	$(GAT)_{11}$	160

Table B. Agronomic performance and percentage of disease incidences (PDI) of the 128 finger millet genotypes; the data was collected after 45 and 90 days of sowing the seeds for PDI and agronomic traits respectively

Name of genotype	PDI (%)	Plant height (cm)	Number of tillers / plant	Number of productive tillers / plan	Number of fingers / head	Length of fingers (cm)	Length of leaf (cm)	Length of root (cm)	Total seed yield (gm)	Cumulative rank	Empirical classification
APSSK1	18.4	63.67	1.00	1.00	5.66	3.67	18.80	14.83	1.50	873.5	S + PAT
CO11	34.4	81.00	2.33	1.67	5.66	7.33	25.67	18.67	2.25	510	S + PAT
CO12	21.6	70.00	2.67	2.00	5.00	7.03	26.33	21.17	1.21	513.5	S + PAT
CO14	3.2	80.30	1.96	1.44	7.30	7.50	34.00	19.00	5.50	299	R +GAT
CO7	24	58.50	2.00	1.67	4.33	3.87	23.67	16.50	1.30	793.5	S + PAT
CO9	35.2	65.00	2.00	2.00	5.33	6.40	28.00	16.67	1.80	638	S + PAT
CONO1	21.6	71.00	3.33	3.00	5.67	7.30	27.83	17.83	2.05	414	S + PAT
DPI00904	42.4	85.00	2.67	2.00	5.00	6.90	32.67	14.00	3.54	481	S + PAT
GPU26	20.8	66.20	1.67	1.33	4.67	5.23	25.00	18.17	1.45	737	S + PAT
GPU28	4	106.60	2.21	2.00	6.30	9.10	39.30	22.60	3.19	207.5	R +GAT
GPU45	15.2	110.30	2.67	1.67	5.67	8.30	25.67	18.17	1.38	428	S + PAT
GPU46	17.6	66.33	2.67	2.33	6.33	6.93	26.67	16.17	1.64	509	S + PAT
GPU48	15.2	44.33	1.33	1.33	3.67	4.50	20.67	18.33	6.30	716	S + PAT
GPU66	6.4	56.33	1.33	1.33	5.00	6.07	21.00	15.03	3.42	700.5	S + PAT
GPU67	4.8	34.33	2.33	1.33	3.67	3.37	18.50	11.03	8.00	752.5	R +PAT
HOSUR1	39.2	47.33	3.33	0.67	3.33	3.83	32.83	20.37	3.25	627.5	S + PAT
HR374	99.6	80.59	3.00	2.22	7.00	10.10	33.00	17.89	5.80	325.5	S + GAT
HR911	67.2	40.00	1.00	1.00	3.67	5.27	16.33	17.70	2.60	899.5	S + PAT

Name of genotype	PDI (%)	Plant height (cm)	Number of tillers / plant	Number of productive tillers / plan	Number of fingers / head	Length of fingers (cm)	Length of leaf (cm)	Length of root (cm)	Total seed yield (gm)	Cumulative rank	Empirical classification
IE1055	44.8	79.83	1.33	1.33	5.33	5.83	22.67	16.83	1.20	782	S + PAT
IE2034	38.4	42.33	3.00	3.00	5.00	4.50	37.00	17.33	1.20	599	S + PAT
IE2042	44	71.67	3.33	3.00	7.00	6.50	22.17	23.23	2.14	432	S + GAT
IE2043	56	69.00	2.33	2.00	8.00	4.40	20.67	16.50	1.25	705.5	S + PAT
IE2217	67.2	71.67	1.67	1.67	7.00	6.17	27.33	18.43	3.00	577.5	S + PAT
IE2296	51.2	62.33	2.33	2.00	6.33	6.33	30.53	16.47	1.25	636	S + PAT
IE2312	52.8	70.67	1.67	1.00	4.00	5.70	22.50	16.83	2.43	800	S + PAT
IE2430	54.4	76.33	1.67	1.67	5.33	5.23	28.17	13.67	1.20	798	S + PAT
IE2437	32	98.67	1.67	1.33	4.67	5.20	31.33	17.17	2.41	614	S + PAT
IE2457	64	99.33	3.33	0.67	1.00	1.80	38.17	17.87	2.35	640	S + PAT
IE2572	55.2	57.33	1.33	2.00	1.20	1.70	44.33	24.40	1.50	740	S + PAT
IE2589	44.8	80.53	2.33	1.67	4.33	5.67	25.67	19.67	3.13	574	S + PAT
IE2606	24.8	64.67	2.67	1.67	4.33	5.67	38.67	14.50	2.13	611	S + PAT
IE2619	38.4	83.00	2.67	1.67	4.33	3.30	31.00	23.33	0.85	613.5	S + PAT
IE2710	16	56.67	3.00	1.68	1.20	1.70	45.00	11.67	3.47	612	S + PAT
IE2790	25.6	86.67	3.33	1.68	1.20	1.70	44.33	19.50	4.45	434	S + PAT
IE2821	63.2	99.00	2.67	1.67	5.67	4.50	33.33	18.33	1.97	527	S + PAT
IE2871	14.4	73.33	2.33	0.67	1.67	2.50	43.87	18.00	2.44	606.5	S + PAT
IE2872	8	86.33	1.67	1.67	6.00	4.53	33.67	17.83	2.41	482.5	S + PAT
IE2911	16.8	52.00	2.67	4.00	7.00	6.17	40.33	15.67	3.11	386.5	S + PAT
IE2957	20	86.00	3.33	3.00	6.50	6.17	46.33	19.00	2.75	250.5	S + GAT

Name of genotype	PDI (%)	Plant height (cm)	Number of tillers / plant	Number of productive tillers / plan	Number of fingers / head	Length of fingers (cm)	Length of leaf (cm)	Length of root (cm)	Total seed yield (gm)	Cumulative rank	Empirical classification
IE3045	20	89.00	1.33	1.33	4.00	7.00	25.87	15.67	1.55	686.5	S + PAT
IE3077	20	91.33	1.33	1.33	4.67	4.83	31.00	16.83	3.11	597.5	S + PAT
IE3104	41.6	54.00	2.33	2.33	9.33	5.23	31.00	16.57	2.75	541.5	S + PAT
IE3317	31.2	53.00	2.00	0.67	3.33	3.67	31.00	16.83	3.33	749.5	S + PAT
IE3391	30.4	50.00	2.67	1.33	4.33	4.00	31.67	15.00	1.36	763.5	S + PAT
IE3392	49.6	56.67	1.67	1.67	5.33	4.03	28.50	22.17	2.38	685.5	S + PAT
IE3470	14.4	77.00	5.00	4.00	7.00	6.17	29.33	15.23	4.30	327	S + GAT
IE3475	18.4	65.33	2.67	1.00	5.00	6.43	28.67	12.63	4.20	594	S + PAT
IE3614	48.8	65.67	2.67	2.33	6.33	6.50	25.17	16.67	2.50	549.5	S + PAT
IE3618	25.6	62.33	2.33	2.00	6.33	5.68	23.00	14.17	3.20	608	S + PAT
IE3721	30.4	56.00	1.67	2.45	2.48	5.65	43.00	13.83	4.50	616.5	S + PAT
IE3945	40.8	60.33	1.33	1.00	2.67	1.80	33.00	15.17	1.50	895.5	S + PAT
IE3952	28.8	66.00	1.67	2.47	3.00	4.50	42.53	19.50	1.70	605	S + PAT
IE3973	33.6	77.67	2.33	2.33	4.33	4.20	31.67	17.17	1.95	592.5	S + PAT
IE4028	18.4	82.67	2.67	2.33	7.00	6.33	31.77	17.67	2.05	374.5	S + PAT
IE4057	21.6	60.33	2.00	2.33	7.00	6.33	32.37	19.00	1.70	476	S + PAT
IE4073	36.8	87.67	2.00	1.33	3.33	4.83	32.80	14.90	2.60	669	S + PAT
IE4121	10.4	104.67	2.33	2.33	4.67	5.43	28.67	19.67	1.64	449	S + PAT
IE4329	17.6	103.67	2.00	1.67	4.33	7.03	28.83	22.00	1.28	488.5	S + PAT
IE4491	16	98.53	2.67	2.33	5.00	7.17	25.67	18.83	2.30	395	S + PAT
IE4497	12	96.00	1.67	1.67	4.00	7.53	30.33	17.83	4.00	440.5	S + PAT

Name of genotype	PDI (%)	Plant height (cm)	Number of tillers / plant	Number of productive tillers / plan	Number of fingers / head	Length of fingers (cm)	Length of leaf (cm)	Length of root (cm)	Total seed yield (gm)	Cumulative rank	Empirical classification
IE4545	43.2	82.33	2.00	1.33	6.00	6.67	27.33	15.33	2.00	639	S + PAT
IE4565	21.6	65.33	2.33	1.00	5.33	7.50	30.50	14.50	3.75	569.5	S + PAT
IE4570	11.2	79.00	2.00	1.67	6.67	4.50	24.20	14.33	4.20	567	S + PAT
IE4622	28	87.33	1.67	1.00	5.00	6.03	24.67	19.00	1.56	666	S + PAT
IE4646	28.8	80.00	2.33	1.33	4.67	6.30	25.13	17.00	3.63	572	S + PAT
IE4671	19.2	99.00	3.00	2.67	7.67	6.50	36.80	14.33	2.35	325.5	S + PAT
IE4673	12.8	82.00	3.00	1.67	4.33	4.50	32.67	17.00	2.80	477	S + PAT
IE4709	28.8	70.00	3.67	3.00	6.33	8.00	32.00	18.67	5.33	276.5	S + GAT
IE4734	2	78.33	1.67	1.33	3.67	3.67	23.67	20.83	2.85	624	R +PAT
IE4757	5.6	104.67	2.00	1.67	6.00	7.33	31.67	18.00	4.00	319	S + PAT
IE4795	1	106.00	3.67	3.00	7.00	9.00	41.00	16.67	3.90	138	R +GAT
IE4797	45.6	45.00	1.67	3.00	7.00	9.00	33.33	15.17	2.75	529	S + PAT
IE4816	41.6	43.00	1.67	3.00	7.00	9.00	36.33	15.57	2.85	498.5	S + PAT
IE501	38.4	55.67	1.33	1.33	3.67	3.67	28.83	19.90	2.17	774	S + PAT
IE5066	33.6	66.67	2.33	1.33	3.67	3.67	36.67	12.67	2.13	734	S + PAT
IE5091	22.4	65.67	2.33	0.33	1.33	1.83	32.33	15.67	1.28	807	S + PAT
IE5106	15.2	84.67	3.00	0.67	1.67	1.80	33.67	17.17	1.72	626.5	S + PAT
IE518	42.4	69.83	2.00	2.00	7.00	4.57	25.03	15.97	2.35	653.5	S + PAT
IE5201	28.8	92.00	3.67	3.33	7.67	4.83	23.00	16.83	2.19	436	S + PAT
IE5306	28	67.00	1.33	1.33	4.33	5.03	20.67	16.50	1.90	816.5	S + PAT
IE5367	21.6	46.33	2.00	1.00	4.33	6.00	20.33	16.00	3.43	741.5	S + PAT

Name of genotype	PDI (%)	Plant height (cm)	Number of tillers / plant	Number of productive tillers / plan	Number of fingers / head	Length of fingers (cm)	Length of leaf (cm)	Length of root (cm)	Total seed yield (gm)	Cumulative rank	Empirical classification
IE5537	33.6	59.33	2.33	1.33	5.67	6.00	22.33	14.17	2.55	715	S + PAT
IE5817	36.8	54.67	2.67	0.33	1.00	1.67	30.67	15.30	3.70	784.5	S + PAT
IE5870	44.8	67.00	1.00	1.00	5.00	3.33	20.67	16.17	1.25	926.5	S + PAT
IE6059	47.2	75.00	2.67	2.67	7.00	7.50	31.00	17.47	2.33	418	S + GAT
IE6082	34.4	73.00	2.00	1.67	4.67	3.83	24.67	11.83	1.73	800	S + PAT
IE6154	7.2	73.00	2.67	2.00	6.00	5.67	25.67	14.50	1.91	561	S + PAT
IE6165	4.8	83.33	2.33	2.33	6.67	6.33	30.00	16.50	2.05	418.5	R +PAT
IE6221	32.8	99.00	2.00	1.67	7.33	5.33	33.33	14.43	2.04	536.5	S + PAT
IE6240	35.2	77.33	2.00	2.00	5.00	5.73	32.00	16.33	1.17	638.5	S + PAT
IE6294	39.2	100.00	3.33	2.67	6.67	6.47	31.67	20.33	1.19	372.5	S + PAT
IE6326	8	103.67	4.33	3.67	7.00	5.30	33.00	15.50	1.65	345.5	S + PAT
IE6337	13.6	74.67	2.00	1.33	5.33	5.50	31.00	20.33	1.20	579.5	S + PAT
IE6350	26.4	89.00	2.33	1.67	7.00	3.50	27.33	21.83	1.89	509	S + PAT
IE6421	13.6	96.33	1.67	1.67	4.67	5.50	27.67	16.23	3.22	552.5	S + PAT
IE6473	21.6	89.00	2.67	1.67	6.33	6.57	34.33	14.67	1.27	481.5	S + PAT
IE6514	24	83.67	2.67	1.33	6.67	6.40	31.00	15.00	1.55	544	S + PAT
IE6537	32	68.00	2.00	1.33	6.87	6.40	34.33	17.67	4.20	473	S + PAT
IE7018	10.4	76.33	3.00	0.67	1.67	1.83	40.33	12.67	1.95	674.5	S + PAT
IE7079	1.5	81.67	2.67	1.00	2.33	2.67	26.67	12.50	2.00	721.5	R +PAT
IE7320	17.6	100.00	3.00	2.67	6.00	5.33	26.67	15.40	2.06	455.5	S + PAT
INDOF5	76.8	57.87	1.00	1.00	4.00	5.67	19.40	15.17	2.90	903.5	S + PAT

Name of genotype	PDI (%)	Plant height (cm)	Number of tillers / plant	Number of productive tillers / plan	Number of fingers / head	Length of fingers (cm)	Length of leaf (cm)	Length of root (cm)	Total seed yield (gm)	Cumulative rank	Empirical classification
INDOF7	24	60.00	2.00	0.33	1.33	3.17	32.17	13.50	2.15	834.5	S + PAT
INDOF8	69.6	75.00	2.33	2.33	4.67	6.17	26.33	19.33	2.79	557.5	S + PAT
INDOF9	68	77.17	2.33	2.00	5.67	6.83	33.83	18.00	3.55	443.5	S + GAT
KM252	70.4	103.60	2.26	2.00	5.60	5.90	30.10	18.10	4.00	477	S + PAT
KM301	73.6	73.67	2.00	1.33	5.00	6.50	26.33	16.33	1.90	727.5	S + PAT
KRI00701	17.6	108.00	2.00	2.00	7.00	6.80	26.67	15.33	3.83	400.5	S + PAT
KRI1311	27.2	64.67	2.00	2.00	5.00	5.57	23.67	14.50	2.17	711	S + PAT
L5	48	69.33	1.00	1.00	5.67	5.97	31.00	18.83	3.15	646	S + PAT
ML365	58.4	92.17	1.67	1.33	5.33	7.30	27.00	19.17	1.78	607.5	S + PAT
MR1	51.2	82.00	1.67	1.33	5.00	7.57	29.53	17.50	3.20	572.5	S + PAT
MR2	59.2	69.00	3.00	2.00	6.00	8.00	42.67	16.07	3.00	438.5	S + GAT
MR6	51.2	80.50	1.33	1.33	6.00	7.17	29.50	19.67	1.10	635.5	S + PAT
PAIYUR2	11.2	110.30	2.30	2.3	6.00	6.4	26.3	19.5	2.46	369	S + PAT
PES110	65.6	99.33	3.00	2.33	6.00	7.00	32.00	17.87	2.65	381.5	S + GAT
PR202	39.2	69.00	1.67	1.33	5.67	6.00	28.33	14.33	2.50	711.5	S + PAT
RAU8	81.6	84.60	2.04	1.30	5.60	6.70	29.30	16.60	8.40	566.5	S + PAT
SVK1	53.6	93.17	3.33	2.33	5.33	9.70	35.20	25.33	5.70	256.5	S + GAT
TCHIN1	47.2	71.67	2.33	0.33	1.67	1.83	41.67	18.33	2.65	685.5	S + PAT
TCUM1	15.2	75.67	3.67	2.00	4.33	5.67	26.33	16.83	2.75	499.5	S + PAT
THRP1	40.8	65.67	1.67	1.33	8.33	4.67	30.50	15.83	1.25	728.5	S + PAT
THRVP	20.8	88.33	1.00	1.00	3.33	6.30	28.33	17.17	3.10	637.5	S + PAT

Name of genotype	PDI (%)	Plant height (cm)	Number of tillers / plant	Number of productive tillers / plan	Number of fingers / head	Length of fingers (cm)	Length of leaf (cm)	Length of root (cm)	Total seed yield (gm)	Cumulative rank	Empirical classification
THRVPP	31.2	88.67	1.67	0.67	3.00	5.10	39.83	19.00	2.41	618	S + PAT
TRY1	38.4	49.00	2.00	1.33	4.67	6.83	22.67	17.83	2.50	697.5	S + PAT
VIJAYAWADA	71.2	70.33	1.33	1.00	6.00	5.70	34.33	20.90	1.80	660	S + PAT
VL149	58.4	71.00	2.00	2.00	7.67	7.17	26.33	16.90	1.65	584.5	S + PAT
VR708	61.6	45.33	1.67	1.67	6.67	4.23	22.17	20.00	2.83	704	S + PAT

PDI- 0% is highly resistant, 1% is resistant, 1.1-5% is moderately resistant, 5.1-25% is moderately susceptible, 25.1 to 50% is susceptible and 50.1 to 100% is highly susceptible. Data on agronomic traits were recorded from 3 representative plants randomly from each row for each genotype and mean value was calculated. Based on empirical classification; three genotypes are resistance with good agronomic traits (R + GATs), ten genotypes are susceptible with good agronomic traits (S + GATs), four genotypes are resistance with poor agronomic traits (R + PATs), and 111 genotypes are susceptible and poor agronomic traits (S + PATs).
